# Dissecting the Native Architecture and Dynamics of Cyanobacterial Photosynthetic Machinery

**DOI:** 10.1016/j.molp.2017.09.019

**Published:** 2017-11-06

**Authors:** Selene Casella, Fang Huang, David Mason, Guo-Yan Zhao, Giles N. Johnson, Conrad W. Mullineaux, Lu-Ning Liu

**Affiliations:** 1 Institute of Integrative Biology, University of Liverpool, Crown Street, Liverpool L69 7ZB, UK; 2 Centre for Cell Imaging, University of Liverpool, Crown Street, Liverpool L69 7ZB, UK; 3 College of Life Science, Shandong Normal University, Jinan 250014, P. R. China; 4 School of Earth and Environmental Sciences, University of Manchester, Oxford Road, Manchester M13 9PT, UK; 5 School of Biological and Chemical Sciences, Queen Mary University of London, Mile End Road, London E1 4NS, UK

**Keywords:** cyanobacteria, thylakoid membrane, atomic force microscopy, photosynthesis, fluorescence imaging, protein dynamics

## Abstract

The structural dynamics and flexibility of cell membranes play fundamental roles in the functions of the cells, i.e., signaling, energy transduction, and physiological adaptation. The cyanobacterial thylakoid membrane represents a model membrane that can conduct both oxygenic photosynthesis and respiration simultaneously. In this study, we conducted direct visualization of the global organization and mobility of photosynthetic complexes in thylakoid membranes from a model cyanobacterium, *Synechococcus elongatus* PCC 7942, using high-resolution atomic force, confocal, and total internal reflection fluorescence microscopy. We visualized the native arrangement and dense packing of photosystem I (PSI), photosystem II (PSII), and cytochrome (Cyt) *b*_6_*f* within thylakoid membranes at the molecular level. Furthermore, we functionally tagged PSI, PSII, Cyt *b*_6_*f*, and ATP synthase individually with fluorescent proteins, and revealed the heterogeneous distribution of these four photosynthetic complexes and determined their dynamic features within the crowding membrane environment using live-cell fluorescence imaging. We characterized red light-induced clustering localization and adjustable diffusion of photosynthetic complexes in thylakoid membranes, representative of the reorganization of photosynthetic apparatus in response to environmental changes. Understanding the organization and dynamics of photosynthetic membranes is essential for rational design and construction of artificial photosynthetic systems to underpin bioenergy development. Knowledge of cyanobacterial thylakoid membranes could also be extended to other cell membranes, such as chloroplast and mitochondrial membranes.

## Teaser

The thylakoid membrane is the site for photosynthetic reactions and responses in cyanobacteria, algae and higher plants. Here, we performed direct visualization of the native organization and mobility of photosynthetic complexes in cyanobacterial thylakoid membranes, using atomic force, confocal, and total internal reflection fluorescence microscopy. Knowledge of the cyanobacterial thylakoid membrane could be extended to chloroplast and mitochondrial membranes.

## Introduction

Oxygenic photosynthesis, the conversion of sunlight into chemical energy by higher plants, green algae, and cyanobacteria, underpins the survival of virtually all higher life forms. Cyanobacteria are the oldest oxygenic phototrophs on Earth. In the cyanobacterial cell, oxygenic photosynthesis typically takes place in the specialized intracellular membranes, namely thylakoid membranes, analogous to higher plants. The cyanobacterial photosynthetic machinery embedded in thylakoid lipid bilayers typically consists of a series of membrane-integral multi-subunit complexes, including photosystem I (PSI), photosystem II (PSII), cytochrome (Cyt) *b*_6_*f*, and ATP synthase (ATPase) complexes. Several small electron transport molecules, such as plastoquinones, plastocyanins, and Cyt *c*_6_, serve as electron carriers to shuttle electrons between individual photosynthetic complexes and promote physiological coordination (DeRuyter and Fromme, 2008). Cyanobacterial thylakoid membranes also act as the site that harbors the components of respiratory electron transport chains, comprising type-I NAD(P)H dehydrogenase-like complex (NDH-1), succinate dehydrogenase (SDH), Cyt oxidase, and alternative oxidase (Vermaas, 2001, Liu et al., 2012, Lea-Smith et al., 2013, Mullineaux, 2014). Remarkable macromolecular crowding and close protein–protein contacts within the cyanobacterial thylakoid membrane result in the dense packing of photosynthetic components (Kirchhoff, 2008, Liu, 2016). While substantial information about the structures and functions of individual photosynthetic components has been available, the spatial organization and dynamics of photosynthetic complexes in the cyanobacterial thylakoid membrane have yet to be well understood experimentally.

The heterogeneity in the composition and localization of photosynthetic proteins in cyanobacterial thylakoid membranes has been reported in previous studies using proteomics, immunoelectron microscopy, and fluorescence microscopy based on their native fluorescent properties (Sherman et al., 1994, Vermaas et al., 2008, Agarwal et al., 2010, Collins et al., 2012). Using hyperspectral confocal fluorescence microscopy, previous studies showed the physical segregation of photosynthetic complexes in the cyanobacterium *Synechocystis* sp. PCC 6803 (*Synechocystis* 6803): the enrichment of PSI in the inner thylakoid regions and the preferential localization of phycobilisomes and PSII in the peripheral thylakoid layers (Vermaas et al., 2008, Collins et al., 2012). By contrast, results from immunoelectron microscopy indicated that the outer thylakoid layer of the cyanobacterium *Synechococcus* sp. PCC 7942 (Syn7942) contains mainly ATPase and PSI, whereas PSII and Cyt *b*_6_*f* are located in both the outer and inner thylakoid layers (Sherman et al., 1994). Nevertheless, it appears that the cyanobacterial thylakoid membrane possesses confined regions that allow for the accommodation and coordination of different photosynthetic components. The lateral segregation of thylakoid membranes could be functionally imperative to the enhancement of photosynthetic performance.

The architecture of cyanobacterial thylakoid membranes is highly dynamic (Mullineaux, 2004, Stingaciu et al., 2016), which is of paramount importance for the formation and maintenance of functional photosynthetic machinery including *de novo* synthesis, turnover and repair of photosynthetic complexes, as well as crosstalk between components. Confocal fluorescence microscopy and fluorescence recovery after photobleaching (FRAP) have been performed to visualize the mobility of photosynthetic complexes in cyanobacterial thylakoid membranes (Mullineaux, 2004). The major supramolecular light-harvesting antenna, phycobilisomes, were shown to be mobile on the stromal surface of the thylakoid membrane (Mullineaux et al., 1997). It was further demonstrated that phycobilisome mobility is required for state transitions (Joshua and Mullineaux, 2004) and non-photochemical quenching (Joshua et al., 2005). In contrast, the membrane-integral PSII complexes exhibit much restricted lateral mobility within the thylakoid membrane, as illustrated by tracking chlorophyll fluorescence (Sarcina et al., 2006); whereas lipid molecules and the IsiA, another chlorophyll-binding membrane protein that is postulated to bind with photosystems and respond to iron deficiency, were determined to be mobile in the thylakoid membrane (Sarcina et al., 2003, Sarcina and Mullineaux, 2004). It is conceivable that the protein organization and specified membrane environment play important roles in determining the diffusion dynamics of photosynthetic complexes in the thylakoid membrane.

Here, we present a direct observation of the native arrangement of photosynthetic complexes in isolated thylakoid membranes from the model cyanobacterium Syn7942, using high-resolution atomic force microscopy (AFM). We also functionally tagged PSI, PSII, Cyt *b*_6_*f*, and ATPase complexes, respectively, with fluorescent proteins and performed live-cell total internal reflection fluorescence (TIRF) microscopy imaging, confocal microscopy imaging, and FRAP analysis to characterize the *in vivo* distribution and mobility fingerprints of these photosynthetic complexes in Syn7942. Our results provide new insights into the compartmentalization and organizational dynamics of the cyanobacterial photosynthetic membrane. Advanced understanding of the architecture and regulation of the photosynthetic machinery exploited in nature is indispensable to the design of artificial photosynthetic systems for improving bioenergy production and manipulation of plant photosynthesis for enhanced agricultural productivity.

## Results

### AFM Topography of Native Thylakoid Membranes from Syn7942

**Figure 1:**
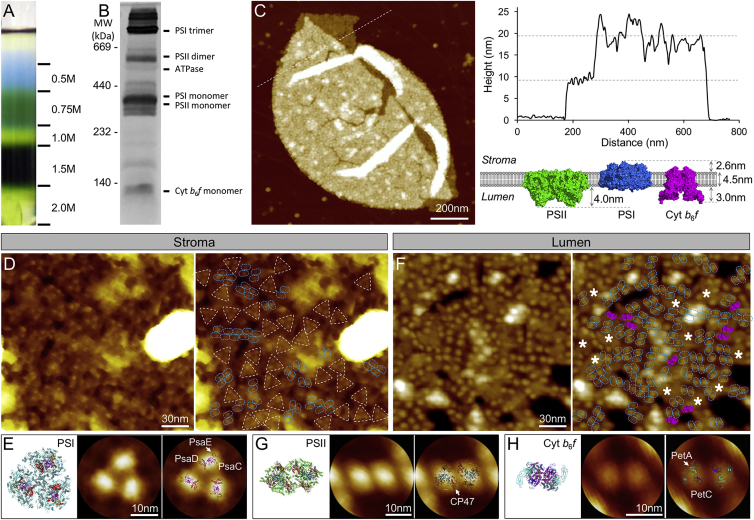
Isolation and AFM Imaging of Native Thylakoid Membranes from Syn7942. **(A)** Step sucrose gradient centrifugation of thylakoid membranes. The 1.5-M fraction containing most of the Chl was extracted for further analysis. **(B)** The blue-native gel was stained with Coomassie blue. Molecular markers are indicated to the left, and the assignment of the major protein complexes is given to the right, based on Zhang et al. (2004). **(C)** AFM topography of isolated thylakoid membrane fragments in liquid solution. It is manifest that photosynthetic membrane complexes are densely packed in the thylakoid membrane. The cross-section profile of thylakoid membranes, along the dashed line, reveals the height of thylakoid membranes. The structure model of the thylakoid membrane (side view) containing dimeric PSII (PDB: 3WU2), trimeric PSI (PDB: 1JB0), and dimeric Cyt *b*_6_*f* (PDB: 4H13). The thylakoid lipid bilayer is about 4.5 nm thick. Analysis of the crystal structures indicates that PSII and Cyt *b*_6_*f* protrude from the lumenal membrane surface by 4.0 nm and 3.0 nm, respectively, whereas the protrusion of PSI complexes from the stromal membrane surface is 2.6 nm high. **(D)** High-resolution AFM image of the stromal surface of thylakoid membranes in liquid solution (left). Individual trimers (white triangles) and dimers (blue ovals) are deduced to be PSI and PSII complexes (right). **(E)** Atomic structure of the PSI complexes from the stromal surface (left, PDB: 1JB0); the three-fold symmetrized correlation average AFM topograph of PSI from the stromal surface of thylakoid membranes (middle, see details in Methods); AFM topograph of PSI superimposed with its atomic structure (right). The protruded subunits of PSI from the stromal surface of thylakoid membranes, PsaC, PsaD, and PsaE, are labeled. **(F)** High-resolution AFM image of the lumenal surface of thylakoid membranes in liquid solution (left). Individual dimers are speculated to be PSII (blue ovals ) and Cyt *b*_6_*f* (pink shadings) complexes (right), based on their protrusions from the lumenal membrane surface (right). White asterisks indicate the possible location of PSI trimers. **(G)** Atomic structure of PSII from the lumenal surface (left, PDB: 3WU2); the two-fold symmetrized correlation average topograph of PSII from the stromal surface (middle); AFM topograph of PSII superimposed with its atomic structure (right). The CP47 subunit of PSII is labeled. **(H)** Atomic structure of Cyt *b*_6_*f* from the lumenal surface (left, PDB: 4H13); the two-fold symmetrized correlation average topograph of Cyt *b*_6_*f* from the stromal surface (middle); AFM topograph of Cyt *b*_6_*f* superimposed with its atomic structure (right). The PetA and PetC subunits of Cyt *b*_6_*f* are labeled.

To study the native organization of cyanobacterial thylakoid membranes, we isolated thylakoid membranes from wild-type Syn7942 cells grown in liquid cultures through cell breakage by glass beads and step sucrose gradient centrifugation in the absence of detergents. Thylakoid membranes were collected from the 1.0–1.5 M fraction (Figure 1A) and were subjected to blue-native polyacrylamide gel electrophoresis (BN-PAGE) characterization of intrinsic photosynthetic complexes (Figure 1B). BN-PAGE reveals the structural integrity of isolated thylakoid membranes, which comprises PSI monomers and trimers (Kruip et al., 1994, El-Mohsnawy et al., 2010), PSII monomers and dimers, Cyt *b*_6_*f*, and ATPase, consistent with the previous result (Zhang et al., 2004). We then conducted AFM imaging in solution to describe the large-scale organization of photosynthetic complexes in the isolated thylakoid membranes. AFM imaging in buffer provides a powerful means of studying biological samples under near-physiological conditions (Liu and Scheuring, 2013). Figure 1C shows an AFM topograph of isolated thylakoid fragments with two membrane bilayers. It is manifest that the thylakoid membrane contains a high content of membrane proteins. Cross-section analysis illustrates that the heights of the single and double thylakoid membranes are 9.49 ± 0.40 nm (*n* = 5) and 18.01 ± 1.10 nm (*n* = 5), respectively. The thickness of a single thylakoid membrane spans the height of the lipid bilayer (4.5 nm), the stromal protrusion of PSI (2.6 nm), and the lumenal protrusion of PSII (4.0 nm) (Jordan et al., 2001, Umena et al., 2011, Engel et al., 2015). High-resolution AFM imaging on the large stacking thylakoid membranes enables us to determine the orientation and long-range distribution of photosynthetic complexes in thylakoid membranes from both stromal and lumenal surfaces. On the stromal surface, trimeric structures (white triangles) were predominately observed, together with some dimeric features (light blue ovals) (Figure 1D). The distribution of these complexes appears relatively random rather than in regular patterns. AFM can acquire data with a lateral resolution of approximately 1 nm and a vertical resolution of 0.1 nm on membrane proteins (Liu and Scheuring, 2013). The three-fold symmetrized correlation average AFM topograph of the trimeric structure matches well the stromal side structure of PSI complexes (Figure 1E, PDB: 1JB0). The three protrusions of PSI trimers are separated by 10.65 ± 0.46 nm (*n* = 60), consistent with previous results (Kruip et al., 1997, Jordan et al., 2001) (Supplemental Figure 1). The surface protrusion of trimeric structures from the membrane bilayer on the stromal side is 2.66 ± 0.25 nm (*n* = 30, Supplemental Figure 1), in close agreement with the vertical dimension of PSI complexes (Figure 1C) (Kruip et al., 1993). Thus, these trimeric structures observed on the stromal surface are assigned to be PSI trimeric complexes. The dimeric objects, often forming arrays, are likely PSII dimers.

By contrast, dimeric structures were mostly seen on the lumenal surface of cyanobacterial thylakoid membranes (Figure 1F). Surface protrusion analysis further revealed that these dimers can be divided into two groups, one of which has a higher vertical protrusion (3.82 ± 0.30 nm, *n* = 60, light blue) from the surface than the other (3.07 ± 0.27 nm, *n* = 20, purple) (Supplemental Figure 1), consistent with the vertical dimensions of PSII and Cyt *b*_6_*f*, respectively (Figure 1C). Two-fold symmetrized correlation average AFM topographs were overlaid with the top-view structures of PSII (Figure 1G, PDB: 3WU2) and Cyt *b*_6_*f* complexes (Figure 1H, PDB: 4H13). The analysis suggests that the dimers with a higher vertical protrusion are PSII dimers, whereas the less-protruded dimeric features are tentatively identified as Cyt *b*_6_*f* proteins, reminiscent of the PSII and Cyt *b*_6_*f* structures observed in spinach grana thylakoids using AFM (Johnson et al., 2014, Phuthong et al., 2015). It is worth noting that, given the less than 1.0-nm difference in the vertical protrusions of PSII and Cyt *b*_6_*f* particles and the architectural complexity on the lumenal surface, we could not accurately identify Cyt *b*_6_*f* complexes in Figure 1F. There are also other particles that we could not easily determine due to the lack of unique topographic structures, i.e., monomers of PSI, PSII, and Cyt *b*_6_*f* complexes, ATPases, and respiratory complexes. Nonetheless, AFM images of both surfaces of cyanobacterial thylakoid membranes illustrate explicitly a crowding membrane environment, with proteins occupying about 75% of the thylakoid membrane area, comparable with the protein-occupied grana thylakoid area in higher plants (Tremmel et al., 2003).

### Distribution of Photosynthetic Complexes in Cyanobacterial Thylakoid Membranes

To study the spatial organization of photosynthetic complexes in cyanobacteria, we tagged PSI, PSII, Cyt *b*_6_*f*, and ATPase individually by fusing the gene for enhanced green fluorescent protein (eGFP) to the 3′ end of genes encoding photosynthetic protein subunits, at the native chromosomal loci of genes and under the control of their endogenous promoters. This ensures that the proteins are expressed in context and at physiological levels. The tagged subunits are PsaE of PSI, CP47 (PsbB) of PSII, Cyt *f* (PetA) of Cyt *b*_6_*f*, and the subunit β (AtpB) of ATPase (Supplemental Figure 2 and Supplemental Table 1). In all cases, the transformants were fully segregated in all copies of the Syn7942 chromosome, confirmed by PCR (Supplemental Figure 2). Immunoblotting analysis using anti-GFP antibody demonstrated that eGFP was fused to the proteins of the expected size (Supplemental Figure 2). BN-PAGE and in-gel fluorescence detection further showed that the eGFP-fused proteins were structurally incorporated into fully assembled photosynthetic multi-subunit complexes (Supplemental Figure 3). Cell growth was not significantly affected by eGFP fusion (Supplemental Figure 4). Moreover, no significant changes in the contents of photosynthetic proteins (absorption and 77K fluorescence spectra, Supplemental Figure 4) and photosynthetic performance (P_700_^+^ re-reduction, apparent F_v_/F_m_, and oxygen evolution, Supplemental Figure 5) were detected, indicating little perturbation of function by eGFP tagging.

**Figure 2:**
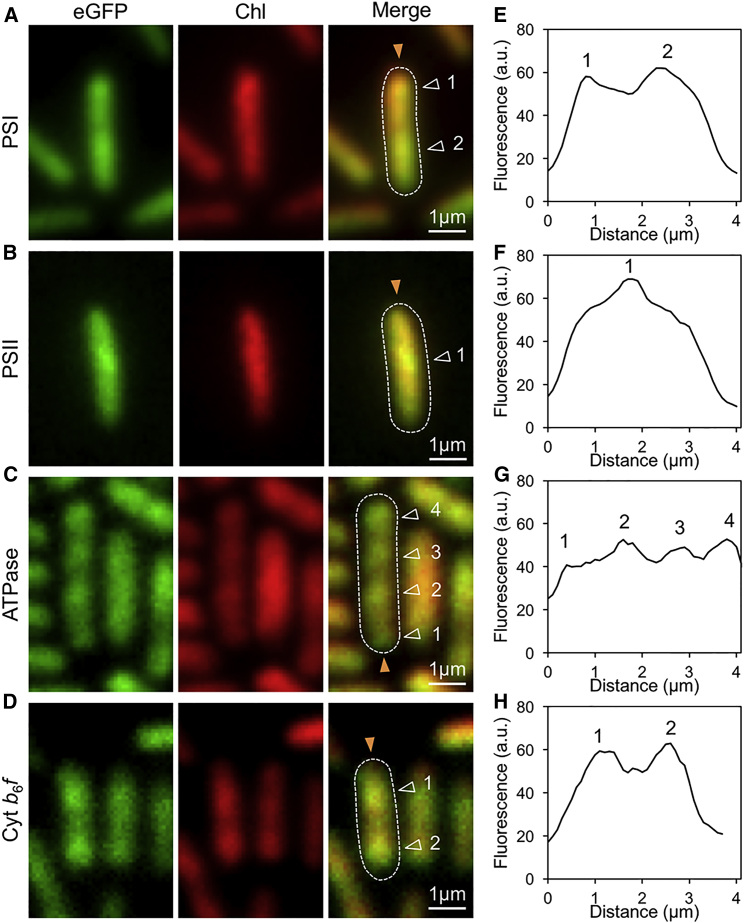
Localization of Photosynthetic Complexes in Syn7942 by TIRF Microscopy. Distribution of photosynthetic complexes in Syn7942 was visualized by near-TIRF microscopy. **(A–D)** Single near-TIRF image frames (150-ms exposure) of PSI:eGFP, PSII:eGFP, ATPase:eGFP, and Cyt *b*_6_*f*:eGFP Syn7942 cells. The GFP and Chl fluorescence were recorded simultaneously. In the merged channel, the distinct fluorescent patches are indicated (white triangles) and cell body borders are outlined based on bright-field images (white dashed lines). **(E–H)** Normalized fluorescence profiles of GFP-tagged cells, taken along the orange arrows indicated in **(A)** to **(D)**.

We visualized eGFP distribution in live Syn7942 cells from exponentially growing cultures using TIRF microscopy (Figure 2). TIRF provides a means to restrict the excitation and detection of fluorophores only near the thin region of cell membranes adherent to the glass surface (about 100 nm thick) while minimizing fluorescence from intracellular regions. It not only offers a higher signal-to-noise ratio and spatial resolution compared with conventional confocal microscopy, but also allows for the visualization of thylakoid membranes from only one side of the cell (with a bias to the outer membrane layers), which potentially facilitates the exploration of heterogeneous protein distribution in the membrane. While eGFP fluorescence was seen throughout the cell membranes, all four eGFP-fused cells presented heterogeneous distribution of photosynthetic complexes (Figure 2). For example, the PSI-eGFP cell shown in Figure 2A has two large membrane patches and the PSII-eGFP cell in Figure 2B possesses one observable membrane patch in the middle of the cell. More distinct spots of fluorescence were observed in the ATPase-eGFP (Figure 2C) and Cyt *b*_6_*f*-eGFP (Figure 2D) cells, indicating the uneven distribution of ATPase and Cyt *b*_6_*f* complexes. Fluorescence profile analysis along the longitudinal axis of the cell verifies the lateral segregation of photosynthetic complexes, representing the specific compartmentalization of cyanobacterial thylakoid membranes (Figure 2E–2H).

**Figure 3:**
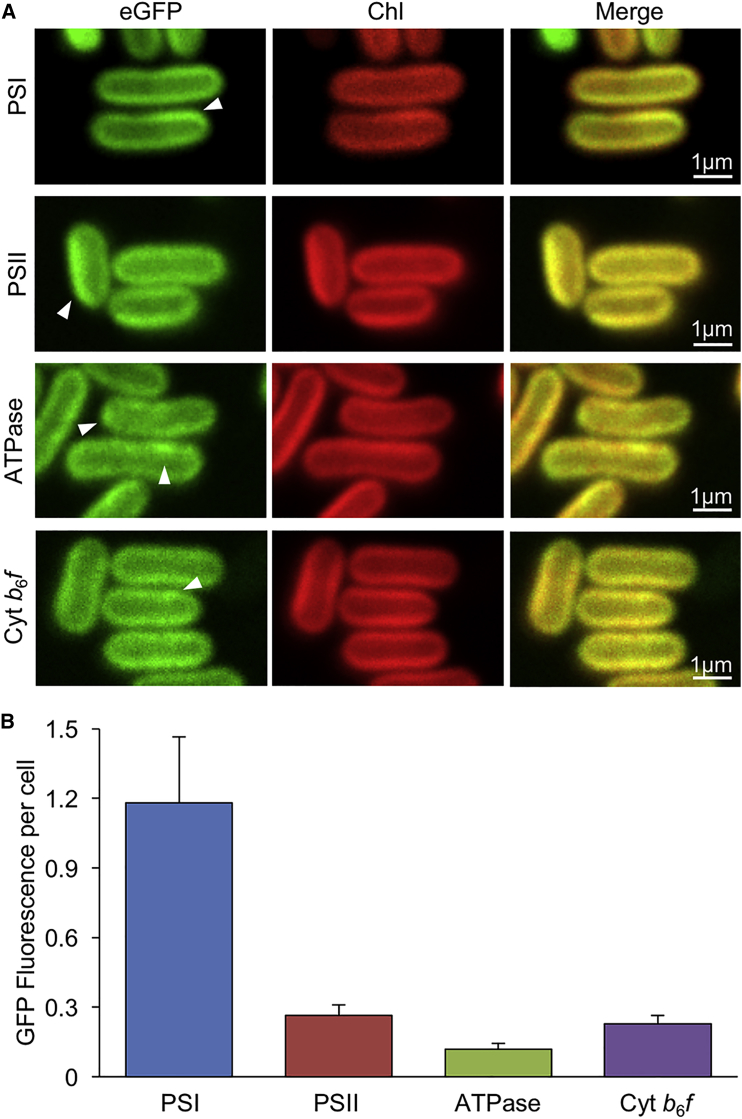
Distribution of Photosynthetic Complexes in Syn7942 by Confocal Microscopy. **(A)** Confocal microscopy images of PSI:eGFP, PSII:eGFP, ATPase:eGFP, and Cyt *b*_6_*f*:eGFP Syn7942 cells. Fluorescence spots of photosynthetic complexes, as indicated by white arrowheads, illustrate the heterogeneous distribution of photosynthetic complexes in thylakoid membranes. **(B)** Quantification of total GFP fluorescence intensities per cell of PSI:eGFP (*n* = 100), PSII:eGFP (*n* = 120), ATPase:eGFP (*n* = 120), and Cyt *b*_6_*f*:eGFP (*n* = 120) strains as in **(A)**. Data are presented as the mean ± SD. See also Supplemental Figure 7.

Using confocal fluorescence microscopy, we further characterized the global distribution of photosynthetic complexes in Syn7942 (Figure 3). The pinhole was set up to obtain a roughly 2.0-μm optical section in the z direction. The four photosynthetic complexes (GFP fluorescence) were located in thylakoid membranes (Chl fluorescence), confirming the functional tagging. The distribution inhomogeneity of the four photosynthetic complexes in whole cells was also observed (Figure 3A, arrows). PSI and PSII complexes tend to aggregate into large membrane regions. By contrast, the ATPase and Cyt *b*_6_*f* complexes exhibit more spotty distribution, in line with TIRF results (Figure 2). The inhomogeneity of GFP fluorescence was further quantified using line profile analysis along the thylakoid membrane and SD calculation, as previously described (Liu et al., 2012). The PSI, ATPase, and Cyt *b*_6_*f* complexes exhibit relatively higher inhomogeneity of the lateral distribution in thylakoid membranes, in contrast to PSII (Supplemental Figure 6).

Moreover, the physiological tagging of eGFP allows us to assess the stoichiometry of photosynthetic complexes at the single-cell level. The relative abundance of GFP molecules per cell was estimated by measuring the brightness of GFP fluorescence in different eGFP-fused cells imaged with the same microscope settings after background subtraction (Figure 3B and Supplemental Figure 7). The PSI/PSII ratio is calculated to be 4.47 (Table 1), slightly higher than the previous data based on the spectroscopic analysis (Joshua and Mullineaux, 2005). This could be ascribed to the variable PSI content under different growth conditions. The PSII/Cyt *b*_6_*f* ratio is 1.16, in good agreement with the previous estimation of 1.0–1.38 (Fujita and Murakami, 1987, Fraser et al., 2013). The PSII/ATPase ratio is 2.25, similar to the ratio examined from immunoblotting results of *Synechocystis* 6803 (Fraser et al., 2013).

**Table 1:** Diffusion Coefficient, Mobile Proportion, and Stoichiometry of Photosynthetic Complexes per Cell Measured Using Confocal Fluorescence Microscopy and FRAP in This Work.

	Diffusion coefficient (*D*, × 10^−10^ cm^2^ s^−1^)	Mobile proportion (%)	Complex ratio	
PSI	0.83 ± 0.27 (*n* = 35)	60 ± 17 (*n* = 30)	PSI/PSII	4.47
PSII	0.98 ± 0.27 (*n* = 45)	75 ± 12 (*n* = 45)	PSI/ATPase	10.06
ATPase	0.83 ± 0.29 (*n* = 30)	76 ± 18 (*n* = 35)	PSI/Cyt *b*_6_*f*	4.76
Cyt *b*_6_*f*	1.62 ± 0.91 (*n* = 30)	78 ± 15 (*n* = 30)	PSII/ATPase	2.25
			PSII/Cyt *b*_6_*f*	1.16
			Cyt *b*_6_*f*/ATPase	1.93

### Mobility of Photosynthetic Complexes in Cyanobacterial Thylakoid Membranes

**Figure 4:**
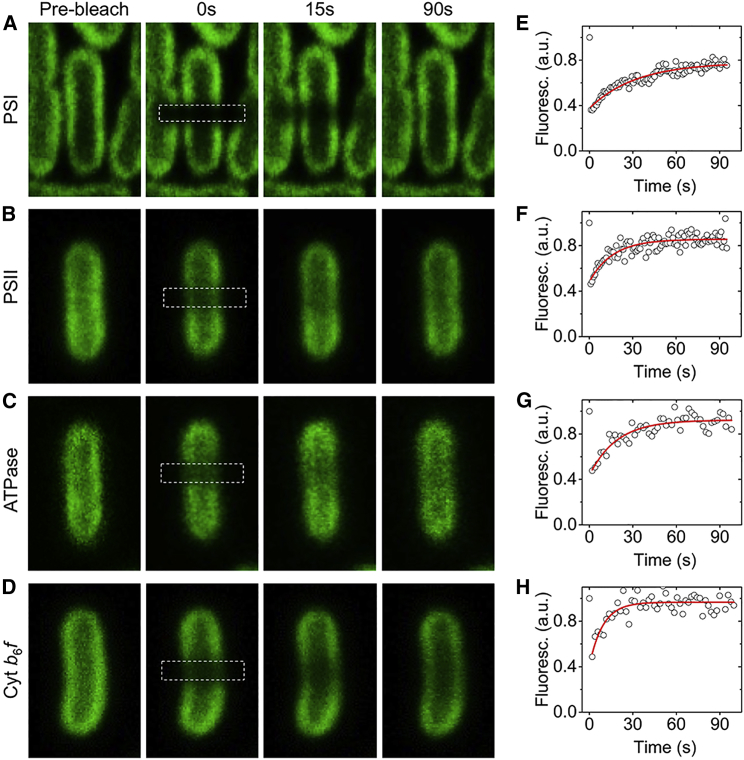
FRAP Analysis of GFP Fluorescence in Individual GFP-Tagged Syn7942 Cells. **(A–D)** Representative FRAP sequence images of PSI:eGFP, PSII:eGFP, ATPase:eGFP, and Cyt *b*_6_*f*:eGFP cells. **(E–H)** Time course of fluorescence recovery of the bleached cell regions (white squares as shown in **A–D**) for GFP fluorescence. Fluorescence values are relative to fluorescence prior to bleaching. The recovery of GFP fluorescence is presented as circles and fitted to an exponential function (red lines).

We further investigated the diffusion dynamics of photosynthetic complexes in the eGFP-fused cells using confocal FRAP measurement with 488-nm excitation. Syn7942 cells have regular and elongated thylakoid membranes that form a set of concentric cylinders aligned along the long axis of the cell, representing an ideal system for FRAP analysis (Mullineaux and Sarcina, 2002). During FRAP measurements, cells were immobilized on the surface of BG11 agar plates at 30°C. The confocal laser spot was used to bleach a line across the center of the cell, using 100% laser power (Figure 4A). For pre- and post-scanning, 7% of the laser power was applied and eGFP fluorescence was detected between 500 and 520 nm. Figure 4A–4D show typical FRAP image sequences of eGFP fluorescence in the four eGFP-labeled strains. Diffusion of photosynthetic components, monitored by repeatedly imaging the same cell and recording the spread and recovery of the bleached line, leads to partial fluorescence recovery in the bleached zone (Mullineaux and Sarcina, 2002, Mullineaux, 2004). The fluorescence recovery curves in the bleached cellular region are depicted in Figure 4E–4H (open circles) and adequately fitted to a single exponential function (red lines).

**Figure 5:**
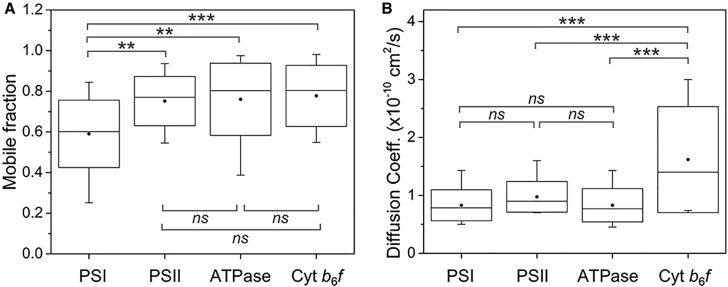
Mobility Features of Photosynthetic Complexes in Thylakoid Membranes of Syn7942. **(A)** Quantification of the mobile fractions of PSI (*n* = 30), PSII (*n* = 45), ATPase (*n* = 35), and Cyt *b*_6_*f* (*n* = 30) in individual GFP-fused cells within 90 s after photobleaching. **(B)** Quantification of the diffusion coefficients of PSI (*n* = 30), PSII (*n* = 45), ATPase (*n* = 35), and Cyt *b*_6_*f* (*n* = 30) in individual GFP-fused cells. Data are presented as mean ± SD. *P* values were calculated using Scheffe's test as indicated: **0.001 < *P* < 0.001, ***P < 0.001; *ns*, not significant.

By tracking the evolution of fluorescence intensity in the bleached regions, we investigated in depth the *in vivo* mobility features of photosynthetic complexes, including the mobile/immobile proportions within 90 s and mean diffusion coefficients of mobile complexes (Figures 4 and 5; Table 1). A common characteristic of the photosynthetic membrane is the exceptional macromolecular crowding (Kirchhoff, 2008, Liu and Scheuring, 2013, Liu, 2016). The high density of photosynthetic membrane complexes in cyanobacterial thylakoid membranes was confirmed by our AFM analysis (Figure 1). Interestingly, our FRAP data reveal that large fractions of photosynthetic complexes are mobile within the crowding membrane environment in 90 s (Figure 5A). PSII (75% ± 12%, *n* = 45), ATPase (76% ± 18%, *n* = 35), and Cyt *b*_6_*f* (78% ± 15%, *n* = 30) complexes exhibit relatively higher mobile fractions than PSI (60% ± 17%, *n* = 30), probably ascribed to the larger dimension of trimeric PSI particles (Table 1).

The diffusion coefficients (*D*), derived from one-dimensional diffusion measurements, represent the average diffusion rates of photosynthetic membrane components over micron-scale distances (Mullineaux, 2004). We found that the PSI, PSII, and ATPase particles have similar *D* (Figure 5B), which are roughly consistent with LHCII antenna's *D* in grana membranes (Kirchhoff et al., 2008). By contrast, the Cyt *b*_6_*f*'s *D* presents a one-fold increase compared with those of the other three photosynthetic components (Figure 5B) and is comparable with that of the Cyt *c* in mitochondrial membranes (Hochman et al., 1982). All four photosynthetic membrane-integrated complexes exhibit significantly reduced diffusion rates than lipids in the thylakoid membranes of Syn7942 (Sarcina et al., 2003).

### Red Light Triggers the Reorganization of Cyanobacterial Photosynthetic Apparatus

**Figure 6:**
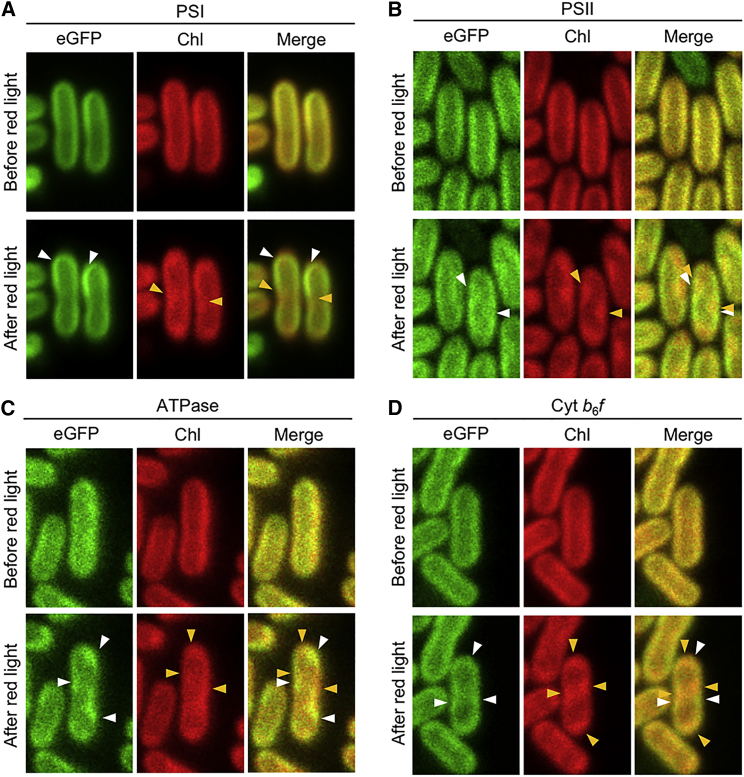
Reorganization of Photosynthetic Complexes Triggered by Intense Red Light. GFP-fused Syn7942 cells (**A**, PSI; **B**, PSII; **C**, ATPase; **D**, Cyt *b*_6_*f*) were illuminated by intense red light of 633 nm for 1.5 min. GFP fluorescence before and after red light treatment was recorded. Patchy organization of eGFP-tagged photosynthetic complexes and chlorophyll fluorescence after red light are indicated by white and orange arrowheads, respectively. Profile analysis of GFP and chlorophyll fluorescence of the GFP-tagged transformants are shown in Supplemental Figure 8.

How the thylakoid membrane organization is modulated in response to environmental stress is still enigmatic. It has been shown that intense red light can trigger the mobility and redistribution of Chl fluorescence to specific zones within the thylakoid membranes, referring to the reorganization and mobility of PSII complexes in Syn7942 (Sarcina et al., 2006). Here, we explored precisely the effects of red light on the distribution and diffusion of four photosynthetic components using the eGFP-tagged strains. Cells were exposed to red light by illuminating a field of 22 × 22 μm using a 633-nm laser (4.76 × 10^7^ μE m^−2^ s^−1^) for 1.5 min. eGFP and Chl fluorescence of the four eGFP-fused Syn7942 cells were detected in the regions of 500–520 nm and 670–720 nm, respectively. After red light treatment, Chl fluorescence was enriched in localized zones within thylakoid membranes (Figure 6, Chl channels, arrowheads), consistent with the previous observation (Sarcina et al., 2006). eGFP channels show specifically the redistribution of individual photosynthetic components triggered by red light. PSI complexes concentrate into one large membrane patch on one end of the cell (Figure 6A). PSII spots were often observed at the center of the cell, reminiscent of the uneven distribution of PSII complexes seen by TIRF (Figure 2B and 2F). Significant spotty zones of ATPases in thylakoid membranes were visualized (Figure 6C). The ATPase membrane “domains” and Chl fluorescence intersperse (Figure 6C, merged channel), suggesting to some extent the independent localization patterns of ATPase and PSII complexes. Similar segregation was also observed for Cyt *b*_6_*f* (Figure 6D).

**Figure 7:**
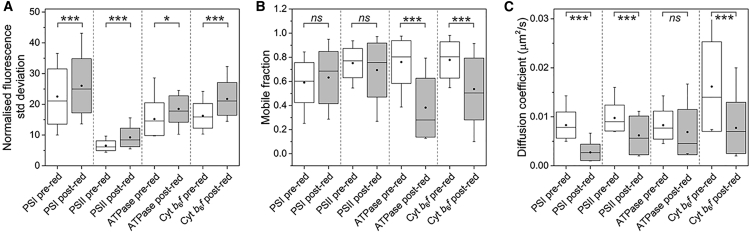
Quantification of the Organization and Mobility of Photosynthetic Complexes before and after Red Light Treatment. **(A)** Normalized fluorescence profile SDs of GFP-tagged cells before and after red light treatment. **(B)** Quantification of the mobile fractions of photosynthetic complexes before and after red light treatment. **(C)** Quantification of the diffusion coefficients of photosynthetic complexes before and after red light treatment. Data are presented as mean ± SD. μg ml^−1^*ns*, not significant.

Our results reveal the presence of specific membrane domains for distinct photosynthetic complexes within the thylakoid membrane. Red light can trigger the redistribution of these photosynthetic components and further segregation into the specific membrane “zones”. This notion is supported by our fluorescence profile analysis (Figure 7A and Supplemental Figure 8). To unravel the diffusion dynamics of photosynthetic complexes after redistribution triggered by red light, we performed FRAP measurement on the cells directly following red light treatment. As shown in Figure 7B, the proportions of mobile PSI and PSII particles appear to be comparable before and after red light treatment, whereas the mobile fractions of ATPase and Cyt *b*_6_*f* complexes are reduced by about 50% and 36%, respectively. In addition, red light led to the decrease of the diffusion coefficients of PSI, PSII, and Cyt *b*_6_*f* complexes by 65%, 34%, and 49%, respectively (Figure 7C). It is plausible that after red light treatment a large proportion of these photosynthetic complexes are concentrated and immobilized into specific membrane regions. In contrast, the diffusion coefficient of ATPase complexes is not significantly affected by red light treatment.

**Figure 8:**
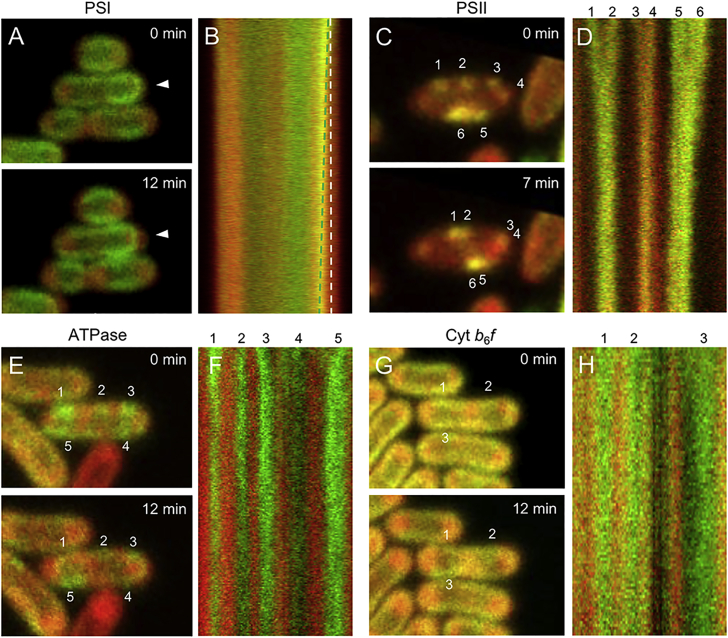
Time-Lapse Confocal Microscopy Imaging of the Redistribution and Dynamics of Photosynthetic Complexes Induced by Red Light. **(A, C, E, and G)** Confocal images of GFP-tagged PSI, PSII, ATPase, and Cyt *b*_6_*f* Syn7942 cells at 0 min and 12 min (7 min for PSII) after red light. White arrowhead in **(A)** indicates the “shrinking” of PSI-eGFP fluorescence profile. See also Supplemental Movies 1, 2, 3, and 4. **(B, D, F, and H)** Kymographs of the distribution of photosynthetic complexes after red light. The green and white dashed lines in **(B)** represent the edges of PSI-eGFP and Chl fluorescence, respectively. Spots of GFP fluorescence in cells were labeled with numbers, corresponding to the spots shown in **(A)**, **(C)**, **(E)**, and **(G)** and were monitored as a function of time.

We further recorded the diffusion dynamics of photosynthetic complexes *in vivo* after red light treatment using time-lapse florescence microscopy (Figure 8). Within 12 min after red light illumination, the PSI-eGFP fluorescence moved toward the cytosol of the cell, in contrast to Chl fluorescence (Figure 8A and Supplemental Movie 1). This is confirmed by the kymograph of eGFP and Chl fluorescence (Figure 8B). By contrast, no significant “shrinking” of the PSII-eGFP fluorescence profile was observable. Instead, the PSII spots were laterally mobile along the thylakoid membrane. It was seen that two distinct PSII spots can merge into one spot within the same membrane patch (Figure 8C and 8D; Supplemental Movie 2). The different redistribution features of PSI and PSII triggered by red light likely indicate the distinct subcellular locations of the two photosynthetic complexes. PSI complexes seem to have a preferential location at the inner layers of cyanobacterial thylakoid membranes, which might be able to bend into the central cytoplasm after red light treatment. Indeed, it has been demonstrated from neutron-scattering experiments that thylakoid membranes in *Synechocystis* 6803 can be quite flexible (Stingaciu et al., 2016). By contrast, PSII complexes are located at the peripheral layers of thylakoid membranes, which have more restricted space for conformational changes. Our results are in line with the previous data acquired from hyperspectral fluorescence microscopy imaging of *Synechocystis* 6803 (Vermaas et al., 2008, Collins et al., 2012). Moreover, ATPase complexes were enriched into specific membrane regions after red light treatment (Figures 8E and 6C). Time-lapse confocal imaging revealed explicitly that these ATPase patches intersperse between Chl fluorescence spots and appear immobile within 12 min (Figure 8F and Supplemental Movie 3). Similarly, no visible mobility of Cyt *b*_6_*f* patches was discerned (Figure 8G and 8H; Supplemental Movie 4). In contrast to the mobile photosynthetic complex patches after red light, the lateral diffusion of photosynthetic complex domains before red light treatment is restricted (Supplemental Figure 9 and Supplemental Movies 5, 6, 7, and 8).

## Discussion

A fundamental question about the cyanobacterial photosynthetic membrane is how their photosynthetic complexes are organized and structurally coordinated in the thylakoid membrane to construct the functional machinery for efficient photosynthetic energy transduction. In this work, we characterized the spatial distribution and mobility of photosynthetic complexes within the cyanobacterial thylakoid membrane using a combination of AFM, TIRF, live-cell confocal microscopy, and FRAP.

The cyanobacterial thylakoid membrane represents an interesting paradigm with highly dense protein arrangement in the membrane lipid bilayer. The components of both photosynthetic and respiratory electron transport chains are accommodated in cyanobacterial thylakoid membranes (Vermaas, 2001, Mullineaux, 2014). Likewise, there are also ion channel proteins within cyanobacterial thylakoid membranes, which are postulated to be key in the balance of trans-thylakoid proton gradient for ATP synthesis (Kuo et al., 2005, Zanetti et al., 2010, Checchetto et al., 2012). Our AFM data reveal that over 75% of the thylakoid membrane surface area is occupied by membrane proteins. This is comparable with the protein densities estimated in grana thylakoids (∼80%) and the whole thylakoid membranes (∼70%) of higher plants and inner mitochondrial membranes (lipid/protein ratio = 0.34) (Ardail et al., 1990, Kirchhoff et al., 2002, Kirchhoff et al., 2004, Tremmel et al., 2003). Moreover, it is notably higher than those of stroma lamellae membranes (∼50%) in higher plants and the plasma membrane (∼23%) (Murphy, 1986, Dupuy and Engelman, 2008). Macromolecular crowding in thylakoid membranes could be essential to retaining strong protein–protein interactions between complexes and a high concentration of chlorophylls to ensure high quantum yields of photosynthetic energy transduction.

Given that many thylakoid complexes possess large dimensions within the lipid bilayer, it is plausible that the lipid space of the cyanobacterial thylakoid membrane will be further restricted. It remains unclear how electron carriers diffuse rapidly in the thylakoid lipids and mediate large-scale electron transport between thylakoid membrane complexes. The pathway of electron flux could be determined by the specific local protein environment and long-range protein organization in membranes, as deduced in purple photosynthetic bacteria (Liu et al., 2009b) and higher plants (Kirchhoff et al., 2002). Our AFM images (Figure 1F) and previous electron microscopy results (Mörschel and Schatz, 1987, Folea et al., 2008) indicate that PSII particles tend to form regular arrays in cyanobacterial thylakoid membranes, which could potentially facilitate the long-range diffusion of small electron carrier molecules. Despite the PSI-enriched domains seen by confocal/TIRF microscopy in the present work and by structured illumination microscopy in a previous study on *Synechocystis* 6803 (MacGregor-Chatwin et al., 2017), we did not observe PSI-only membrane patches as reported recently (MacGregor-Chatwin et al., 2017). This is probably due to the absence of detergent treatment during our membrane preparation, different growth conditions, or the species-dependent variation. By contrast, AFM images show that PSI complexes are intermixed with PSII (Figure 1D). The PSI–PSII membrane regions have also been observed in other cyanobacteria such as *Synechocystis* 6803, *Thermosynechococcus*, and *Synechococcus* sp. PCC 7002 (MacGregor-Chatwin et al., 2017). The integration of PSI and PSII within the same thylakoid membrane regions in cyanobacteria could be structurally fundamental to state transitions, a mechanism for modulating the energy transfer from phycobilisomes to PSI and PSII, balancing the energy captured by phycobilisomes between PSI and PSII in response to changing conditions (Mullineaux and Emlyn-Jones, 2005). This provides the architectural basis for the movement of phycobilisomes between PSI and PSII (Joshua and Mullineaux, 2004) or local conformational changes of phycobilisome-PSI-PSII domains (McConnell et al., 2002).

Previous studies characterizing the large-scale distribution and dynamics of phycobilisomes, PSI, and PSII in cyanobacterial cells were based on the fluorescence of phycobilins and chlorophylls (Mullineaux et al., 1997, Vermaas et al., 2008). However, the extremely high concentration and fluctuation of pigments could largely restrict the sensitivity of fluorescence detection. Intense laser on phycobilins and chlorophylls, which closely interact with each other, may result in intrinsic photoprocesses of these pigments (Liu et al., 2009a). Moreover, accurate distinction of PSI and PSII is challenging based on only chlorophyll fluorescence, because of the notable overlap of PSII and PSII fluorescence, the low and rapid decay of PSI fluorescence at room temperature, and the high PSI/PSII ratio in cyanobacteria (Turconi et al., 1993, Joshua and Mullineaux, 2005, Sarcina et al., 2006). To overcome this, we tagged eGFP to PSI, PSII, Cyt *b*_6_*f*, and ATPase, respectively, and used live-cell TIRF and confocal microscopy to probe their location and diffusion dynamics *in vivo*. Direct detection of eGFP fluorescence allows us to exclude any photochemical processes that occur in photosynthesis, i.e., quenching of fluorescence. All four complexes have inhomogeneous distribution in the thylakoid membrane. Apart from those widespread over the thylakoid membrane, PSI and PSII complexes tend to aggregate within large membrane domains. It was shown that PSI complexes present a higher heterogeneity than PSII, probably implying that PSI complexes are more abundant than others in certain regions of the thylakoid membrane (Supplemental Figure 6). How photosynthetic complexes are organized within these membrane domains awaits further investigation.

To our knowledge, this is the first observation of the patchy distribution and mobile features of Cyt *b*_6_*f* and ATPase complexes in living cyanobacterial cells (Figure 2, Figure 3, Figure 4). Using AFM, we also observed that some tentative Cyt *b*_6_*f* complexes are spread in the PSII domains (Figure 1F). It is unclear whether Cyt *b*_6_*f* are close to PSI, since the two photosynthetic complexes have opposite protrusions above the thylakoid surfaces. No ATPase complexes were identified in AFM images, probably due to the weak association of the whole complex and their specific membrane locations that may not be accessible to the AFM probe (Liu and Scheuring, 2013). Molecular organizations of Cyt *b*_6_*f* and ATPase in the thylakoid membrane require further exploration.

The different distribution patterns of photosynthetic complexes may indicate the functional and structural segregation of thylakoid membrane zones in fulfilling distinct roles in photosynthetic electron transport. In line with the heterogeneous organization of photosynthetic complexes, the respiratory complexes, NDH-1 and SDH, have been reported to present spatial segregation (on the scale of 100–300 nm) within the thylakoid membrane of Syn7942 (Liu et al., 2012). Reorganization of respiratory NDH-1 complexes in thylakoid membranes, triggered by different light intensity, could serve as a physiological mechanism to channel the pathways of electron flow. Taken together, it appears that cyanobacterial thylakoid membranes possess specific domains to accommodate functionally relevant bioenergetic components and compartmentalize metabolism. Partitioning of bioenergetic complexes has also been observed in other membrane systems. For instance, in the primordial cyanobacterium *Gloeobacter violaceus*, which has no internal thylakoid membranes, both the photosynthetic and the respiratory complexes are concentrated in two distinct plasma membrane domains (Rexroth et al., 2011); the photosynthetic components are spatially separated in the thylakoid membranes of higher plants—PSII and light-harvesting complex II in grana stacks with most of the PSI, light-harvesting complex I, and ATPase in unstacked membrane regions (Kirchhoff, 2008); the oxidative phosphorylation enzymes are clustered in specific zones within the plasma membranes of Gram-negative *Escherichia coli* and Gram-positive *Bacillus subtilis* (Johnson et al., 2004, Lenn et al., 2008, Llorente-Garcia et al., 2014); and different respiratory complexes are asymmetrically distributed in the mitochondrial inner membranes (Vogel et al., 2006, Wilkens et al., 2013). The structural and functional segregation of membrane proteins appears to be a general characteristic of any biological membranes.

We characterized the diffusional fingerprints of the four photosynthetic complexes within thylakoid membranes. More than 60% of photosynthetic complexes are mobile in the crowding membrane environment under physiological conditions (Table 1), resembling the mobility of chlorophyll–protein complexes in isolated grana membranes from spinach (Kirchhoff et al., 2008). By monitoring directly eGFP fluorescence, we found that about 75% of PSII are mobile under physiological conditions, with a diffusion coefficient of 0.98 × 10^−10^ cm^2^ s^−1^. This differs from the previous study that suggested the immobility of PSII in Syn7942 by tracking Chl fluorescence (Sarcina et al., 2001). For comparison, we performed FRAP measurements on PSI-eGFP cells, excited with a 633-nm laser, to detect Chl fluorescence recovery (Supplemental Figure 10). The results show greatly restricted Chl diffusion in the same Syn7942 cell that we used for FRAP analysis on eGFP fluorescence, ruling out the possibilities that the mobility of photosynthetic complexes determined by FRAP on eGFP fluorescence arises from different growth conditions or is strain dependent. Functional fluorescence tagging allows us to determine the diffusion coefficients of PSI, ATPase, and Cyt *b*_6_*f* in thylakoid membranes for the first time (PSI 0.83 × 10^−10^ cm^2^ s^−1^, ATPase 0.83 × 10^−10^ cm^2^ s^−1^, Cyt *b*_6_*f* 1.62 × 10^−10^ cm^2^ s^−1^, Table 1). The diffusion rates of PSI, PSII, ATPase, and Cyt *b*_6_*f* are in the same magnitude as the diffusion rates of IsiA in cyanobacterial thylakoid membranes (Sarcina and Mullineaux, 2004) and light-harvesting complex II in both grana and stroma membranes (Consoli et al., 2005, Kirchhoff et al., 2008). However, the diffusion coefficients of cyanobacterial photosynthetic complexes are roughly more than one order of magnitude lower than those of membrane proteins in the eukaryotic plasma membrane (Frick et al., 2007), ER membrane (Nehls et al., 2000), and mitochondrial membrane (Gupte et al., 1991). This further corroborates the high protein density of thylakoid membranes.

The diffusion dynamics of photosynthetic complexes represents the combination of the mobility of photosynthetic complexes within the complex-enriched membrane regions and between the patches. It is feasible that the immobile photosynthetic complexes are concentrated mainly within the discrete membrane zones, whereas the mobile photosynthetic complexes can diffuse between each membrane zone. Recently, increasing experimental evidence has proved the occurrence of photosynthetic/respiratory supercomplexes in cyanobacteria, for instance, PSII–PSI (Bečková et al., 2017), PSI-PSII-phycobilisome (Liu et al., 2013), PSI–phycobilisome (Watanabe et al., 2014), and NDH-1–PSI (Gao et al., 2016). Concomitantly, the bioenergetic supercomplexes have also been characterized in chloroplasts and mitochondria (Iwai et al., 2010, Lapuente-Brun et al., 2013). The observed bioenergetic membrane domains may act as the pools where functionally relevant complexes physically associate to form electron transport supercomplexes. However, a recent study has reported that mobile domains of different oxidative phosphorylation enzymes in the *E. coli* plasma membrane do not co-localize, in contrast to the “respirazones” notion (Llorente-Garcia et al., 2014). Whether the structural association of different photosynthetic complexes in cyanobacteria is transient or dynamic in response to changes in the environmental conditions, as described in the “plasticity model” (Acin-Perez et al., 2008), is a subject for future study, e.g., visualization of pairs of fluorescently tagged photosynthetic complexes.

Our results showed that red light can induce the reorganization of protein complexes in thylakoid membranes, which may represent a combination of protein mobility within membranes and the structural reorganization of thylakoid membranes (Sarcina et al., 2006, Iwai et al., 2015). The finding has categorical implications for the dynamic organization of cyanobacterial thylakoid membranes, which is instrumental in environmental acclimation. The different reorganization features of PSI and PSII complexes, triggered by red light, suggest the non-uniform localization of PSI and PSII in different thylakoid membrane sacs in Syn7942: PSI complexes appear to be located mainly at the inner layers of thylakoid membranes and PSII complexes are likely distributed at the peripheral layers of thylakoid membranes (Figure 8). This is consistent with previous hyperspectral confocal microscopy results obtained from *Synechocystis* 6803 (Vermaas et al., 2008, Collins et al., 2012). We further revealed that red light can reduce the diffusion rates of PSII, as well as PSI and ATPase, while it has an undetectable effect on the mobility of Cyt *b*_6_*f* (Figure 7). Interestingly, the PSII patches show increased mobility after red light (Figure 8), reminiscent of the previous finding that PSII mobility could be induced by red light (Sarcina et al., 2006). Nevertheless, it is evident that the distribution and mobility of photosynthetic complexes in cyanobacterial thylakoid membranes are variable in response to environmental changes, to act as the molecular “tuners” for manipulating the pathways and performance of electron transport in cyanobacteria. Future investigation will focus on elucidating, at the molecular level, how cyanobacteria sense the alternations of natural environmental conditions and manipulate their photosynthetic machinery for optimized energy-transduction performance.

In short, by using the combination of AFM, TIRF, confocal microscopy, and FRAP, our study provides deeper insight into the spatial organization and mobility of photosynthetic complexes in the cyanobacterial thylakoid membrane. The thylakoid membrane possesses a high protein density that could potentially promote electron transduction. Rather than being evenly located within the native thylakoid membrane, the photosynthetic complexes tend to possess clustering distribution and are mobile in thylakoids, suggesting the specific organization and functional compartmentalization of the thylakoid membrane. The membrane localization and diffusion dynamics of photosynthetic complexes can be altered by environmental changes, such as intense red light. The acquired information about cyanobacterial thylakoid membranes will facilitate the understanding of how the photosynthetic machinery is functionally configured and physiologically regulated. It could represent a model system that likely applies to understanding other biological membranes. In translational terms, advanced knowledge about the photosynthetic machinery will inform the rational design of artificial photosynthesis to promote bioenergy production and manipulation of plant photosynthesis to improve the capture and conversion of solar energy into biomass for enhanced agricultural productivity.

## Methods

### Strains and Growth Conditions

*Synechococcus elongatus* PCC 7942 (Syn7942) strains were grown photoautotrophically in BG11 medium (Rippka et al., 1979) at 30°C under constant white illumination (50 μE m^−2^ s^−1^) in culture flasks with constant shaking, or on BG-11 plates containing 1.5% (w/v) agar. eGFP mutants were cultured in the presence of 50 μg ml^−1^ of apramycin. Growth in plate was used both to select transformed colonies and maintenance of GFP-tagged strains. The *E. coli* strains used in this work were DH5α and BW25113. *E. coli* was grown aerobically at 30°–37°C in Luria–Bertani (LB) medium. Medium supplements were used where appropriate at the following final concentrations: ampicillin 100 μg ml^−^^1^, chloramphenicol 10 μg ml^−^^1^, apramycin 50 μg ml^−^^1^, and arabinose 100 μM.

### Generation of Constructs

eGFP fusions were created by inserting the eGFP:apramycin region amplified from the plasmid pIJ786 (PBL Biomedical Laboratories) to the C terminus of *psaE*, *psbB*, *petA*, or *atpB* using the Redirect strategy (Gust et al., 2002, Gust et al., 2004). Plasmids were verified by PCR and used to transform Syn7942 cells following the method described earlier (Golden, 1988). PCR, agarose gel electrophoresis, and immunoblot analysis were applied to check genetic segregation of the *psaE*, *psbB*, *petA*, or *atpB* loci in the transformants. The DNA oligonucleotides used in this work are shown in Supplemental Table 1.

### Thylakoid Membrane Isolation and BN-PAGE

Syn7942 membrane fractions were prepared by glass bead (212–300 μm in diameter) breakage at 4°C followed by centrifugation (Zhang et al., 2004). To obtain pure thylakoid membranes, we further separated the membrane fractions in a step sucrose gradient (2.0 M, 1.5 M, 1.0 M, 0.75 M, 0.5 M) and centrifuged them at 40 000 rpm in Beckman 70Ti for 1 h at 4°C. The Chl-enriched samples at 1.0–1.5 M sucrose step were collected and characterized by high-resolution AFM imaging in buffer (Figure 1). No detergent was added during membrane isolation and AFM imaging to ensure the physiological organization of isolated thylakoid membranes.

For BN-PAGE, isolated membranes were prepared according to the previously described method (Zhang et al., 2004). Similar amounts of protein samples with 6 μg of Chl were loaded on the gels for membrane fraction samples. Membrane protein samples were separated either on 12% (v/v) denaturing SDS–PAGE gels (Laemmli, 1970), or on 3%–12% (v/v) linear gradient native BN-PAGE gels (Zhang et al., 2004). Gels were either stained with Coomassie blue R-250 or electroblotted onto polyvinylidene difluoride (PVDF) membrane or in-gel GFP fluorescence detection (see Supplemental Information).

### Cell Growth, Absorption Spectra, Immunoblotting Analysis, PSII Quantum Efficiency, P_700_^+^ Re-reduction Measurements, 77K Fluorescence Spectra, and Oxygen Evolution

For details, see Supplemental Information.

### Atomic Force Microscopy

Freshly purified thylakoid membranes at 4°C were diluted in adsorption buffer (10 mM Tris–HCl [pH 7.2], 150 mM KCl, 25 mM MgCl_2_). Five microliters of thylakoid membrane samples were adsorbed onto freshly cleaved mica surface with 40 μl of adsorption buffer at room temperature for more than 1 h. After adsorption, the sample was carefully rinsed five times with imaging buffer (10 mM Tris–HCl [pH 7.2], 150 mM KCl). AFM imaging was performed in peak force tapping mode in liquid at room temperature using a Bruker Multimode 8.0 equipped with a J-scanner with NanoScope V controller (Bruker, Santa Barbara, USA). AFM tips with the spring constant of 0.4 N m^−^^1^ (ScanAssyst Air HR; Bruker) were used for high-resolution imaging and the tip spring constant was routinely calibrated. The average imaging force was ∼100 pN. All AFM topographs had an image size of 512 × 512 pixels.

Particle averaging was performed using home-made cross-correlation-based Java routines for the ImageJ image processing package (Scheuring et al., 2007, Fechner et al., 2009, Liu et al., 2011). Photosynthetic complexes have internal symmetry: PSI trimers have three-fold symmetry whereas PSII and Cyt *b*_6_*f* dimers have two-fold symmetry. The topographical image of a single symmetrized PSI trimer (or PSII/Cyt *b*_6_*f* dimer) was used as a reference to calculate correlation maps by comparing the reference with the raw high-resolution image. All identified proteins were averaged and the average was symmetrized for creating the reference for the second and conclusive cross-correlation cycle, to obtain the final three-fold (or two-fold) symmetrized correlation averages of PSI trimers (or PSII/Cyt *b*_6_*f* dimers).

### Total Internal Reflection Fluorescence Microscopy

Syn7942 cells were immobilized on BG11 agar plates as reported previously (Liu et al., 2012). Cells were then imaged with TIRF illumination in a Zeiss LSM880 providing laser excitation at 488 nm and 561 nm for GFP and phycobilins/chlorophyll, respectively, by a 100× oil objective lens (numerical aperture 1.46) and an extra 1.6× magnification. Fluorescence emission was imaged by an Evolve 512 Delta EMCCD Camera (Photometrics, USA). GFP and phycobilins/chlorophyll fluorescence were detected between 510 and 555 nm and 581 and 679 nm, respectively. The focal plane was set at 79 nm from the glass coverslip surface to image the transmembrane surface of the cyanobacterium. The incident angle of the laser was maintained at around 68°. The image exposure time is about 150 ms.

### Confocal Fluorescence Microscopy and Analysis

Live-cell confocal fluorescence imaging was performed on a Zeiss LSM710 or LSM780 inverted confocal microscope with a 100× oil-immersion objective (numerical aperture: 1.45) and excitation at 488 nm. Images (12-bit, 512 × 512 pixels) were recorded by averaging each scan line eight times. The confocal pinhole was set to give z-axis resolution of about 2 μm. GFP and chlorophyll fluorescence were detected at 500–520 nm and 670–720 nm, respectively. Red light treatments were performed by illuminating the cells using a 633-nm laser (4.76 × 10^7^ μE m^−2^ s^−1^) for 1.5 min.

Uneven distribution of the photosynthetic complexes was quantified by taking line profiles of fluorescence intensity around the thylakoid membranes, smoothing to remove high-frequency noise and then computing the SD from the mean fluorescence intensity. GFP fluorescence intensity of cells was determined by measuring the total GFP fluorescence of individual cells and extracting background fluorescence of empty regions with the same area. GFP fluorescence intensity per cell was normalized to the cell length and chlorophyll intensity. Image analysis was undertaken from results of the eGFP-tagged strains of three independent biological repeats. Results are presented as the mean ± SD.

### Fluorescence Recovery after Photobleaching and Analysis

Sample preparation and FRAP experiments were carried out as described previously (Mullineaux, 2004, Sarcina and Mullineaux, 2004). Recovery of fluorescence profiles after photobleaching was monitored for 90 s to ensure the stationary of fluorescence intensity. No reversible recovery of GFP fluorescence was detected after photobleaching the whole cell (data not shown). FRAP data analysis was performed following the previous procedure (Mullineaux et al., 1997, Mullineaux, 2004, Kirchhoff et al., 2008). For comparison of fluorescence distributions before and after bleaching, fluorescence profiles were normalized to the same total fluorescence. The post-bleach profiles were then subtracted from the pre-bleach profile to generate a set of difference profiles. The mobile fraction of GFP-tagged complexes was determined by the following formula:

$$M=\frac{F_{\mathrm{final}}-F_{\mathrm{post}}}{F_{\mathrm{pre}}-F_{\mathrm{post}}}$$

where *M* is the mobile fraction, *F*_final_ is the final fluorescence, *F*_post_ is the post-bleach fluorescence, and *F*_pre_ is the scaled pre-bleach fluorescence.

To estimate the diffusion coefficient, we took the first post-bleach difference profile and used a home-made computer routine based on SigmaPlot 13 (Systat Software, San Jose, USA) to generate a series of predicted fluorescence profiles at various times after the bleaching, assuming an arbitrary diffusion coefficient for random diffusion (Kirchhoff et al., 2008). The predicted fluorescence recovery curve was fitted to the experimentally observed fluorescence recovery curve by adjusting the time axis.

## Funding

L.-N.L. acknowledges a Royal Society University Research Fellowship (UF120411), a Royal Society Research grant for University Research Fellowship (RG130442), a Royal Society Challenge grant (CH160004), and Biotechnology and Biological Sciences Research Council grants (BB/R003890/1 and BB/M024202/1). S.C. acknowledges the PhD scholarship provided by the Institute of Integrative Biology, University of Liverpool. F.H. acknowledges a Leverhulme Trust Early Career Fellowship (ECF-2016-778). G.-Y.Z. acknowledges a Fellowship of Chinese Postdoctoral International Exchange Program (20170058) and a Scholarship for Outstanding Young Teachers of Shandong Province. We acknowledge the Liverpool Center for Cell Imaging for technical assistance and access to confocal/TIRF microscopes (Medical Research Council, MR/K015931/1; Biotechnology and Biological Sciences Research Council, BB/M012441/1).

## Author Contributions

Conceptualization, L.-N.L. and C.W.M; Methodology, S.C., F.H., G.-Y.Z., C.W.M., and L.-N.L.; Investigation, S.C., F.H., and L.-N.L.; Formal Analysis, S.C., D.M., G.N.J., C.W.M., and L.-N.L.; Writing – Original Draft, S.C., F.H., and L.-N.L.; Writing – Review & Editing, G.-Y.Z., D.M., G.N.J., C.W.M., and L.-N.L.; Funding Acquisition, F.H., G.-Y.Z., and L.-N.L.; Resources, D.M. and G.N.J.; Supervision, L.-N.L. and C.W.M.

## Supplemental Information

Document S1. Supplemental Methods, Supplemental Figures 1–10, Supplemental Table 1, and Supplemental Reference

Supplemental Movie 1. Dynamics of PSI Complexes after Red Light Treatment Data were recorded for 12 min and shown at 15 frames per second. Snapshots and kymograph are presented in Figure 8.

Supplemental Movie 2. Dynamics of PSII Complexes after Red Light Treatment Data were recorded for 7 min and shown at 15 frames per second. Snapshots and kymograph are presented in Figure 8.

Supplemental Movie 3. Dynamics of ATPase Complexes after Red Light Treatment Data were recorded for 12 min and shown at 15 frames per second. Snapshots and kymograph are presented in Figure 8.

Supplemental Movie 4. Dynamics of Cyt *b*_6_*f* Complexes after Red Light Treatment Data were recorded for 12 min and shown at 15 frames per second. Snapshots and kymograph are presented in Figure 8.

Supplemental Movie 5. Dynamics of PSI Complexes before Red Light Treatment Data were recorded for 10 min and shown at 15 frames per second. Snapshots and kymograph are presented in Supplemental Figure 9.

Supplemental Movie 6. Dynamics of PSII Complexes before Red Light Treatment Data were recorded for 10 min and shown at 15 frames per second. Snapshots and kymograph are presented in Supplemental Figure 9.

Supplemental Movie 7. Dynamics of ATPase Complexes before Red Light Treatment Data were recorded for 12 min and shown at 15 frames per second. Snapshots and kymograph are presented in Supplemental Figure 9.

Supplemental Movie 8. Dynamics of Cyt *b*_6_*f* Complexes before Red Light Treatment Data were recorded for 12 min and shown at 15 frames per second. Snapshots and kymograph are presented in Supplemental Figure 9.

## References

[bib1] Acin-Perez R., Fernandez-Silva P., Peleato M.L., Perez-Martos A., Enriquez J.A. (2008). Respiratory active mitochondrial supercomplexes. Mol. Cell.

[bib2] Agarwal R., Matros A., Melzer M., Mock H.P., Sainis J.K. (2010). Heterogeneity in thylakoid membrane proteome of *Synechocystis* 6803. J. Proteomics.

[bib3] Ardail D., Privat J.P., Egret-Charlier M., Levrat C., Lerme F., Louisot P. (1990). Mitochondrial contact sites. Lipid composition and dynamics. J. Biol. Chem..

[bib4] Bečková M., Gardian Z., Yu J., Konik P., Nixon P.J., Komenda J. (2017). Association of Psb28 and Psb27 proteins with PSII-PSI supercomplexes upon exposure of *Synechocystis* sp. PCC 6803 to high light. Mol. Plant.

[bib5] Checchetto V., Segalla A., Allorent G., La Rocca N., Leanza L., Giacometti G.M., Uozumi N., Finazzi G., Bergantino E., Szabo I. (2012). Thylakoid potassium channel is required for efficient photosynthesis in cyanobacteria. Proc. Natl. Acad. Sci. USA.

[bib6] Collins A.M., Liberton M., Jones H.D., Garcia O.F., Pakrasi H.B., Timlin J.A. (2012). Photosynthetic pigment localization and thylakoid membrane morphology are altered in *Synechocystis* 6803 phycobilisome mutants. Plant Physiol..

[bib7] Consoli E., Croce R., Dunlap D.D., Finzi L. (2005). Diffusion of light-harvesting complex II in the thylakoid membranes. EMBO Rep..

[bib8] DeRuyter Y.S., Fromme P., Herrero A., Flores E. (2008). Molecular structure of the photosynthetic apparatus. The Cyanobacteria: Molecular Biology, Genomics, and Evolution.

[bib9] Dupuy A.D., Engelman D.M. (2008). Protein area occupancy at the center of the red blood cell membrane. Proc. Natl. Acad. Sci. USA.

[bib10] El-Mohsnawy E., Kopczak M.J., Schlodder E., Nowaczyk M., Meyer H.E., Warscheid B., Karapetyan N.V., Rogner M. (2010). Structure and function of intact photosystem 1 monomers from the cyanobacterium *Thermosynechococcus elongatus*. Biochemistry.

[bib11] Engel B.D., Schaffer M., Kuhn Cuellar L., Villa E., Plitzko J.M., Baumeister W. (2015). Native architecture of the *Chlamydomonas* chloroplast revealed by in situ cryo-electron tomography. Elife.

[bib12] Fechner P., Boudier T., Mangenot S., Jaroslawski S., Sturgis J.N., Scheuring S. (2009). Structural information, resolution, and noise in high-resolution atomic force microscopy topographs. Biophys. J..

[bib13] Folea I.M., Zhang P., Aro E.M., Boekema E.J. (2008). Domain organization of photosystem II in membranes of the cyanobacterium *Synechocystis* PCC6803 investigated by electron microscopy. FEBS Lett..

[bib14] Fraser J.M., Tulk S.E., Jeans J.A., Campbell D.A., Bibby T.S., Cockshutt A.M. (2013). Photophysiological and photosynthetic complex changes during iron starvation in *Synechocystis* sp. PCC 6803 and *Synechococcus elongatus* PCC 7942. PLoS One.

[bib15] Frick M., Schmidt K., Nichols B.J. (2007). Modulation of lateral diffusion in the plasma membrane by protein density. Curr. Biol..

[bib16] Fujita Y., Murakami A. (1987). Regulation of electron transport composition in cyanobacterial photosynthetic system: stoichiometry among photosystem I and II complexes and their light-harvesting antennae and cytochrome *b*_6_/*f* complex. Plant Cell Physiol..

[bib17] Gao F., Zhao J., Chen L., Battchikova N., Ran Z., Aro E.M., Ogawa T., Ma W. (2016). The NDH-1L-PSI supercomplex is important for efficient cyclic electron transport in cyanobacteria. Plant Physiol..

[bib18] Golden S.S. (1988). Mutagenesis of cyanobacteria by classical and gene-transfer-based methods. Methods Enzymol..

[bib19] Gupte S.S., Chazotte B., Leesnitzer M.A., Hackenbrock C.R. (1991). Two-dimensional diffusion of F_1_F_0_-ATP synthase and ADP/ATP translocator. Testing a hypothesis for ATP synthesis in the mitochondrial inner membrane. Biochim. Biophys. Acta.

[bib20] Gust B., Chandra G., Jakimowicz D., Yuqing T., Bruton C.J., Chater K.F. (2004). Lambda red-mediated genetic manipulation of antibiotic-producing *Streptomyces*. Adv. Appl. Microbiol..

[bib21] Gust B., Kieser T., Chater K.F. (2002). REDIRECT Technology: PCR-Targeting System in *Streptomyces coelicolor*.

[bib22] Hochman J.H., Schindler M., Lee J.G., Ferguson-Miller S. (1982). Lateral mobility of cytochrome *c* on intact mitochondrial membranes as determined by fluorescence redistribution after photobleaching. Proc. Natl. Acad. Sci. USA.

[bib23] Iwai M., Takizawa K., Tokutsu R., Okamuro A., Takahashi Y., Minagawa J. (2010). Isolation of the elusive supercomplex that drives cyclic electron flow in photosynthesis. Nature.

[bib24] Iwai M., Yokono M., Nakano A. (2015). Toward understanding the multiple spatiotemporal dynamics of chlorophyll fluorescence. Plant Signal. Behav..

[bib25] Johnson A.S., van Horck S., Lewis P.J. (2004). Dynamic localization of membrane proteins in *Bacillus subtilis*. Microbiology.

[bib26] Johnson M.P., Vasilev C., Olsen J.D., Hunter C.N. (2014). Nanodomains of cytochrome *b*_6_*f* and photosystem II complexes in spinach grana thylakoid membranes. Plant Cell.

[bib27] Jordan P., Fromme P., Witt H.T., Klukas O., Saenger W., Krauss N. (2001). Three-dimensional structure of cyanobacterial photosystem I at 2.5 Å resolution. Nature.

[bib28] Joshua S., Bailey S., Mann N.H., Mullineaux C.W. (2005). Involvement of phycobilisome diffusion in energy quenching in cyanobacteria. Plant Physiol..

[bib29] Joshua S., Mullineaux C.W. (2004). Phycobilisome diffusion is required for light-state transitions in cyanobacteria. Plant Physiol..

[bib30] Joshua S., Mullineaux C.W. (2005). The *rpaC* gene product regulates phycobilisome-photosystem II interaction in cyanobacteria. Biochim. Biophys. Acta.

[bib31] Kirchhoff H. (2008). Molecular crowding and order in photosynthetic membranes. Trends Plant Sci..

[bib32] Kirchhoff H., Haferkamp S., Allen J.F., Epstein D.B., Mullineaux C.W. (2008). Protein diffusion and macromolecular crowding in thylakoid membranes. Plant Physiol..

[bib33] Kirchhoff H., Mukherjee U., Galla H.J. (2002). Molecular architecture of the thylakoid membrane: lipid diffusion space for plastoquinone. Biochemistry.

[bib34] Kirchhoff H., Tremmel I., Haase W., Kubitscheck U. (2004). Supramolecular photosystem II organization in grana thylakoid membranes: evidence for a structured arrangement. Biochemistry.

[bib35] Kruip J., Boekema E.J., Bald D., Boonstra A.F., Rogner M. (1993). Isolation and structural characterization of monomeric and trimeric photosystem I complexes (P700.FA/FB and P700.FX) from the cyanobacterium *Synechocystis* PCC 6803. J. Biol. Chem..

[bib36] Kruip J., Bald D., Boekema E., Rogner M. (1994). Evidence for the existence of trimeric and monomeric Photosystem I complexes in thylakoid membranes from cyanobacteria. Photosynth. Res..

[bib37] Kruip J., Chitnis P.R., Lagoutte B., Rögner M., Boekema E.J. (1997). Structural organization of the major subunits in cyanobacterial photosystem 1. Localization of subunits PsaC, -D, -E, -F, and -J. J. Biol. Chem..

[bib38] Kuo M.M., Haynes W.J., Loukin S.H., Kung C., Saimi Y. (2005). Prokaryotic K^+^ channels: from crystal structures to diversity. FEMS Microbiol. Rev..

[bib39] Laemmli U.K. (1970). Cleavage of structural proteins during the assembly of the head of bacteriophage T4. Nature.

[bib40] Lapuente-Brun E., Moreno-Loshuertos R., Acin-Perez R., Latorre-Pellicer A., Colas C., Balsa E., Perales-Clemente E., Quiros P.M., Calvo E., Rodriguez-Hernandez M.A. (2013). Supercomplex assembly determines electron flux in the mitochondrial electron transport chain. Science.

[bib41] Lea-Smith D.J., Ross N., Zori M., Bendall D.S., Dennis J.S., Scott S.A., Smith A.G., Howe C.J. (2013). Thylakoid terminal oxidases are essential for the cyanobacterium *Synechocystis* sp. PCC 6803 to survive rapidly changing light intensities. Plant Physiol..

[bib42] Lenn T., Leake M.C., Mullineaux C.W. (2008). Clustering and dynamics of cytochrome *bd*-I complexes in the *Escherichia coli* plasma membrane *in vivo*. Mol. Microbiol..

[bib43] Liu L.N. (2016). Distribution and dynamics of electron transport complexes in cyanobacterial thylakoid membranes. Biochim. Biophys. Acta.

[bib44] Liu L.N., Scheuring S. (2013). Investigation of photosynthetic membrane structure using atomic force microscopy. Trends Plant Sci..

[bib45] Liu L.N., Aartsma T.J., Thomas J.C., Zhou B.C., Zhang Y.Z. (2009). FRAP analysis on red alga reveals the fluorescence recovery is ascribed to intrinsic photoprocesses of phycobilisomes than large-scale diffusion. PLoS One.

[bib46] Liu L.N., Duquesne K., Sturgis J.N., Scheuring S. (2009). Quinone pathways in entire photosynthetic chromatophores of *Rhodospirillum photometricum*. J. Mol. Biol..

[bib47] Liu L.N., Sturgis J.N., Scheuring S. (2011). Native architecture of the photosynthetic membrane from *Rhodobacter veldkampii*. J. Struct. Biol..

[bib48] Liu L.N., Bryan S.J., Huang F., Yu J.F., Nixon P.J., Rich P.R., Mullineaux C.W. (2012). Control of electron transport routes through redox-regulated redistribution of respiratory complexes. Proc. Natl. Acad. Sci. USA.

[bib49] Liu H., Zhang H., Niedzwiedzki D.M., Prado M., He G., Gross M.L., Blankenship R.E. (2013). Phycobilisomes supply excitations to both photosystems in a megacomplex in cyanobacteria. Science.

[bib50] Llorente-Garcia I., Lenn T., Erhardt H., Harriman O.L., Liu L.N., Robson A., Chiu S.W., Matthews S., Willis N.J., Bray C.D. (2014). Single-molecule *in vivo* imaging of bacterial respiratory complexes indicates delocalized oxidative phosphorylation. Biochim. Biophys. Acta.

[bib51] MacGregor-Chatwin C., Sener M., Barnett S.F., Hitchcock A., Barnhart-Dailey M.C., Maghlaoui K., Barber J., Timlin J.A., Schulten K., Hunter C.N. (2017). Lateral segregation of photosystem I in cyanobacterial thylakoids. Plant Cell.

[bib52] McConnell M.D., Koop R., Vasil'ev S., Bruce D. (2002). Regulation of the distribution of chlorophyll and phycobilin-absorbed excitation energy in cyanobacteria. A structure-based model for the light state transition. Plant Physiol..

[bib53] Mörschel E., Schatz G.H. (1987). Correlation of photosystem-II complexes with exoplasmatic freeze-fracture particles of thylakoids of the cyanobacterium *Synechococcus* sp. Planta.

[bib54] Mullineaux C.W. (2004). FRAP analysis of photosynthetic membranes. J. Exp. Bot..

[bib55] Mullineaux C.W. (2014). Co-existence of photosynthetic and respiratory activities in cyanobacterial thylakoid membranes. Biochim. Biophys. Acta.

[bib56] Mullineaux C.W., Emlyn-Jones D. (2005). State transitions: an example of acclimation to low-light stress. J. Exp. Bot..

[bib57] Mullineaux C.W., Sarcina M. (2002). Probing the dynamics of photosynthetic membranes with fluorescence recovery after photobleaching. Trends Plant Sci..

[bib58] Mullineaux C.W., Tobin M.J., Jones G.R. (1997). Mobility of photosynthetic complexes in thylakoid membranes. Nature.

[bib59] Murphy D.J. (1986). The molecular organisation of the photosynthetic membranes of higher plants. Biochim. Biophys. Acta.

[bib60] Nehls S., Snapp E.L., Cole N.B., Zaal K.J., Kenworthy A.K., Roberts T.H., Ellenberg J., Presley J.F., Siggia E., Lippincott-Schwartz J. (2000). Dynamics and retention of misfolded proteins in native ER membranes. Nat. Cell Biol..

[bib61] Phuthong W., Huang Z., Wittkopp T.M., Sznee K., Heinnickel M.L., Dekker J.P., Frese R.N., Prinz F.B., Grossman A.R. (2015). The use of contact mode atomic force microscopy in aqueous medium for structural analysis of spinach photosynthetic complexes. Plant Physiol..

[bib62] Rexroth S., Mullineaux C.W., Ellinger D., Sendtko E., Rogner M., Koenig F. (2011). The plasma membrane of the cyanobacterium *Gloeobacter violaceus* contains segregated bioenergetic domains. Plant Cell.

[bib63] Rippka R., Deruelles J., Waterbury J.B., Herdman M., Stanier R.Y. (1979). Generic assignments, strain histories and properties of pure cultures of cyanobacteria. J. Gen. Microbiol..

[bib64] Sarcina M., Mullineaux C.W. (2004). Mobility of the IsiA chlorophyll-binding protein in cyanobacterial thylakoid membranes. J. Biol. Chem..

[bib65] Sarcina M., Tobin M.J., Mullineaux C.W. (2001). Diffusion of phycobilisomes on the thylakoid membranes of the cyanobacterium *Synechococcus* 7942. Effects of phycobilisome size, temperature, and membrane lipid composition. J. Biol. Chem..

[bib66] Sarcina M., Murata N., Tobin M.J., Mullineaux C.W. (2003). Lipid diffusion in the thylakoid membranes of the cyanobacterium *Synechococcus* sp.: effect of fatty acid desaturation. FEBS Lett..

[bib67] Sarcina M., Bouzovitis N., Mullineaux C.W. (2006). Mobilization of photosystem II induced by intense red light in the cyanobacterium *Synechococcus* sp PCC7942. Plant Cell.

[bib68] Scheuring S., Boudier T., Sturgis J.N. (2007). From high-resolution AFM topographs to atomic models of supramolecular assemblies. J. Struct. Biol..

[bib69] Sherman D.M., Troyan T.A., Sherman L.A. (1994). Localization of membrane proteins in the cyanobacterium *Synechococcus* sp. PCC7942 (Radial asymmetry in the photosynthetic complexes). Plant Physiol..

[bib70] Stingaciu L.R., O'Neill H., Liberton M., Urban V.S., Pakrasi H.B., Ohl M. (2016). Revealing the dynamics of thylakoid membranes in living cyanobacterial cells. Sci. Rep..

[bib71] Tremmel I.G., Kirchhoff H., Weis E., Farquhar G.D. (2003). Dependence of plastoquinol diffusion on the shape, size, and density of integral thylakoid proteins. Biochim. Biophys. Acta.

[bib72] Turconi S., Schweitzer G., Holzwarth A.R. (1993). Temperature dependence of picosecond fluorescence kinetics of a cyanobacterial photosystem I particle. Photochem. Photobiol..

[bib73] Umena Y., Kawakami K., Shen J.R., Kamiya N. (2011). Crystal structure of oxygen-evolving photosystem II at a resolution of 1.9 Å. Nature.

[bib74] Vermaas W.F. (2001). Photosynthesis and Respiration in Cyanobacteria. In: Encyclopedia of Life Sciences.

[bib75] Vermaas W.F., Timlin J.A., Jones H.D., Sinclair M.B., Nieman L.T., Hamad S.W., Melgaard D.K., Haaland D.M. (2008). *In vivo* hyperspectral confocal fluorescence imaging to determine pigment localization and distribution in cyanobacterial cells. Proc. Natl. Acad. Sci. USA.

[bib76] Vogel F., Bornhovd C., Neupert W., Reichert A.S. (2006). Dynamic subcompartmentalization of the mitochondrial inner membrane. J. Cell Biol..

[bib77] Watanabe M., Semchonok D.A., Webber-Birungi M.T., Ehira S., Kondo K., Narikawa R., Ohmori M., Boekema E.J., Ikeuchi M. (2014). Attachment of phycobilisomes in an antenna-photosystem I supercomplex of cyanobacteria. Proc. Natl. Acad. Sci. USA.

[bib78] Wilkens V., Kohl W., Busch K. (2013). Restricted diffusion of OXPHOS complexes in dynamic mitochondria delays their exchange between cristae and engenders a transitory mosaic distribution. J. Cell Sci..

[bib79] Zanetti M., Teardo E., La Rocca N., Zulkifli L., Checchetto V., Shijuku T., Sato Y., Giacometti G.M., Uozumi N., Bergantino E. (2010). A novel potassium channel in photosynthetic cyanobacteria. PLoS One.

[bib80] Zhang P., Battchikova N., Jansen T., Appel J., Ogawa T., Aro E.M. (2004). Expression and functional roles of the two distinct NDH-1 complexes and the carbon acquisition complex NdhD3/NdhF3/CupA/Sll1735 in *Synechocystis* sp PCC 6803. Plant Cell.

