# Supplementary material for: Dissecting the Native Architecture and Dynamics of Cyanobacterial Photosynthetic Machinery
Source: Mol Plant. 2017 Nov 6;10(11):1434–48. doi: 10.1016/j.molp.2017.09.019 (PMC5683893; doi:10.1016/j.molp.2017.09.019)
Supplement: Document S1. Supplemental Methods, Supplemental Figures 1–10, Supplemental Table 1, and Supplemental Reference [file mmc1.docx]

**Supplemental Information**

**Dissecting the native architecture and dynamics of cyanobacterial photosynthetic machinery**

Selene Casella^1^, Fang Huang^1^, David Mason^1,2^, Guo-Yan Zhao^1,3^, Giles N. Johnson^4^, Conrad W. Mullineaux^5^, Lu-Ning Liu^1,*^

^1^Institute of Integrative Biology, University of Liverpool, Crown Street, Liverpool L69 7ZB, United Kingdom.

^2^Centre for Cell Imaging, University of Liverpool, Crown Street, Liverpool L69 7ZB, United Kingdom.

^3^College of Life Science, Shandong Normal University, Jinan 250014, P. R. China.

^4^School of Earth and Environmental Sciences, University of Manchester, Oxford Road, Manchester M13 9PT, United Kingdom.

^5^School of Biological and Chemical Sciences, Queen Mary University of London, Mile End Road, London E1 4NS, United Kingdom.

^*^Correspondence: luning.liu@liverpool.ac.uk

**SUPPLEMENTAL METHODS**

**Cell growth and absorption spectra**

Growth of the cells was monitored by turbidity at 750 nm with a Jenway 6300 UV/Vis spectrophotometer (Jenway, UK). Absorbance spectra of intact cells or TMs suspensions were carried out at room temperature through a SpectraMax 340 Microplate Reader (Molecular Devices, USA) between the wavelengths of 400 nm and 750 nm. Chlorophyll a content was determined by extraction into methanol and absorption measurement taken at 666 nm and 750 nm through Jenway 6300 (Jenway, UK), as previously described (Komenda and Barber, 1995).

**Immunoblotting analysis and in-gel GFP fluorescence detection**

Gels were either stained with Coomassie Blue R-250 or electroblotted onto polyvinylidene difluoride (PVDF) membrane (0.2-μm pore size; Bio-Rad Laboratories, UK). Immunoblotting analyses were performed with primary mouse polyclonal anti-GFP antibody diluted 1:5,000, and horseradish peroxidase-conjugated goat anti-mouse immunoglobulin G secondary antibody (GE Healthcare, UK) diluted 1:5,000. Signals were visualized using a chemiluminescence kit (SuperSignal West Pico, Pierce). GFP signal was detected directly in BN-PAGE gels using an ImageQuant LAS 4000 (GE Healthcare Life Sciences, excitation blue epi-RGB, filter detector for GFP). Experiments were done for all the tagged photosynthetic complexes in at least three biological repeats.

**Quantum efficiency of Photosystem II**

Cells grown at the exponential phase were diluted to a chlorophyll concentration of ∼1-2 μg∙mL^-1^ in a volume of 4 ml and dark adapted for three minutes prior to Fv/Fm measurement. Fv/Fm was measured from three biological replicates using an AquaPen-C fluorometer (Photon Systems Instruments, Czech Republic) with an excitation of 620 nm. Experimental data were analyzed using an AquaPen-C software package.

**P700^+^ re-reduction measurements**

Changes in absorbance at 830 nm with labels on different subunits of the major photosynthetic complexes. Cells were illuminated with 5-s pulses of red light (*lmax* = 623 nm; *I* = 2000 µmol∙m^-2^∙s^-1^) repeated at 30-second intervals. Each curve represents the mean of 10 accumulations from each of 2 separate aliquots of cells. Curves are normalized to the absorbance of WT following illumination for 5 s.

**77K fluorescence emission spectra**

Steady-state fluorescence emission spectra at 77 K were measured in intact cells adapted in the dark. Aliquots of cells suspended in BG11 to 5 μM chlorophyll were injected into silica capillary tubes of internal diameter 2.5 mm being frozen in liquid nitrogen. Emission spectra were obtained by placing these tubes in the liquid nitrogen housing of a Perkin Elmer LS50 Luminescence Spectrophotometer (Foster City, CA) equipped with a red-sensitive photomultiplier. Excitation and emission slit widths were set at 5 nm. Fluorescence emission spectra were recorded with the excitation of phycobilins and chlorophyll at 600 nm and 435 nm, respectively.

**Oxygen evolution**

O_2_ evolution of cell cultures was measured in the dark at 30°C after illumination using saturating red light in a Clarke-type oxygen electrode (OxyLab 2, Hansatech, King’s Lynn, UK). One mL of cell suspension with Chl concentration of 20 µM was placed into the electrode chamber, aerated and sealed from the atmosphere.

**Supplementary Reference:**

**Komenda, J., and Barber, J.** (1995). Comparison of *psbO* and *psbH* deletion mutants of *Synechocystis* PCC 6803 indicates that degradation of D1 protein is regulated by the Q_B_ site and dependent on protein synthesis. Biochemistry 34:9625-9631.

**Supplemental Table 1.** PCR Primers

| **Primer** | | **Sequence** |
| --- | --- | --- |
| **PSI** | FpsaE | CGAGGTAGCGCGACAGATAG |
|  | RpsaE | GCCATAGACCCACGCTTGAC |
|  | RFpsaEGFP | GAAGCAGAATTACAAGTGGTTGCAGCAGCCGCCAAAAAACTGCCGGGCCCGGAGCTGCC |
|  | RRpsaEGFP | GTAGCCGTTTCAGGAACCTCTTGTGAAGACAGAAGCAGTATTCCGGGGATCCGTCGACC |
|  | FpsaEseg | CGCGTGGTGATAAGGTTCGG |
|  | RpsaEseg | GAGTAAGACTTCGCCACCTG |
| **PSII** | FCP47 | CTACAAAGCGCTGCGGATGG |
|  | RCP47 | AATCCCGCACGCCTCGAAAC |
|  | RFCP47GFP | CAGAAATTGGGTGACCCGACCACTCGGAAAACAGCCGCTCTGCCGGGCCCGGAGCTGCC |
|  | RRCP47GFP | GAATGAGCATCACCCAAACCGCTCTAACCATTCACAACCATTCCGGGGATCCGTCGACC |
|  | FCP47seg | GCGTGGCTGGTTCACCTTTG |
|  | RCP47seg | GCTGTAGGCCAGTTGTAGCG |
| **Cyt *b*_6_*f*** | FpetA | GTTTGGGCAACTCTATCTGG |
|  | RpetA | CCTGCACTAAAGCTCACTAC |
|  | RFpetAGFP | AAACAAGTCGAGAAAGTGCAAGCTGCTGAGCTGAACTTCCTGCCGGGCCCGGAGCTGCC |
|  | RRpetAGFP | GGCCCGCCCTCTCTCTTGCAACCGTTACTGCAACCAGATATTCCGGGGATCCGTCGACC |
|  | FpetAseg | CGCTGACCAACGATCCGAAC |
|  | RpetAseg | GAGGCCATACCAACGGATGC |
| **ATPase** | FatpB | GAAGCGGCTTGACGAATCAG |
|  | RatpB | TCTCCACCGATGAGTCCTAC |
|  | RFatpBGFP | GAAGCCATCGAAAAAGGCGCCAAGCTGAAAGCTGAATCCCTGCCGGGCCCGGAGCTGCC |
|  | RRatpBGFP | GCGGGGCTGCGATCGCAACCCCACCAAAGTCAGCCAAACATTCCGGGGATCCGTCGACC |
|  | FatpBseg | CCTTCTTCGTGGCTGAAGTG |
|  | RatpBseg | TGACCCGGCAAGATACCGAG |


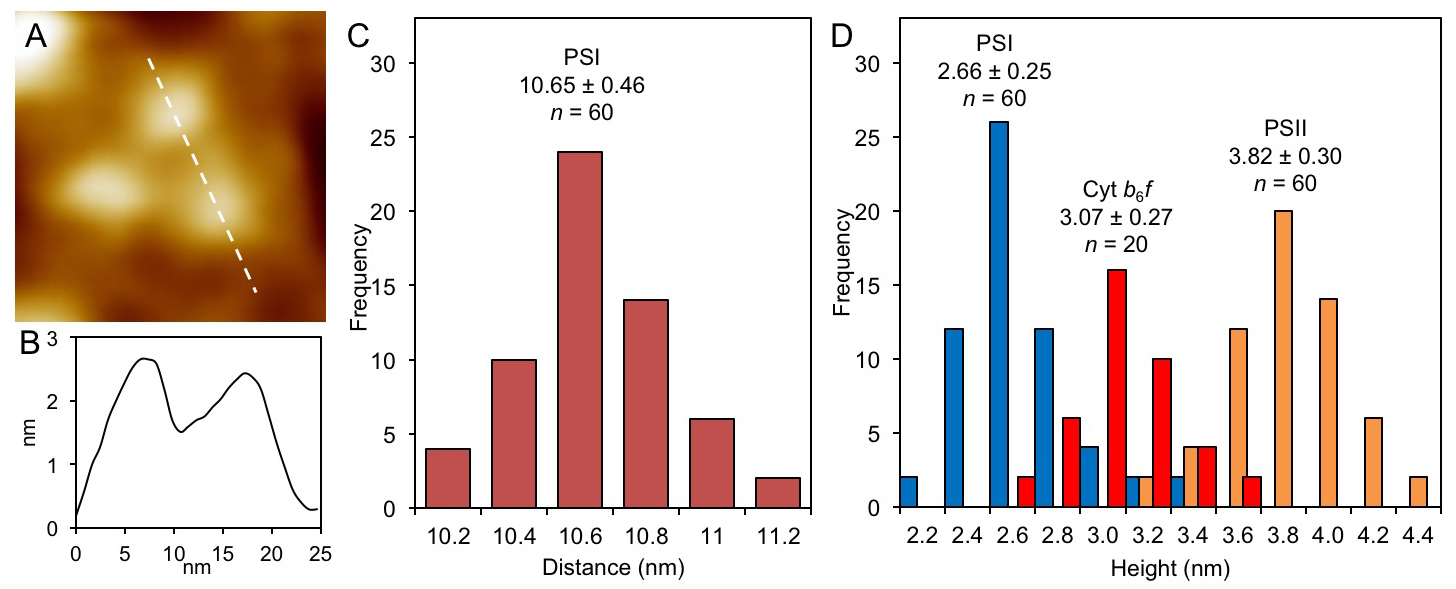


**Supplemental Figure 1. AFM analysis of photosynthetic complexes in native thylakoid membranes.**

(A) High-resolution AFM image of a single trimer, which is assigned to be a PSI complex.

(B) Height profile analysis of the protrusions in the trimeric structure, along the dashed line shown in (A).

(C) Histogram of the lateral distance of monomers within a PSI trimer from the stromal surface of thylakoid membranes, along the dashed line shown in (A).

(D) Histogram of the protrusion heights of PSI, PSII, and Cyt *b*_6_*f* complexes above the thylakoid lipid bilayer.


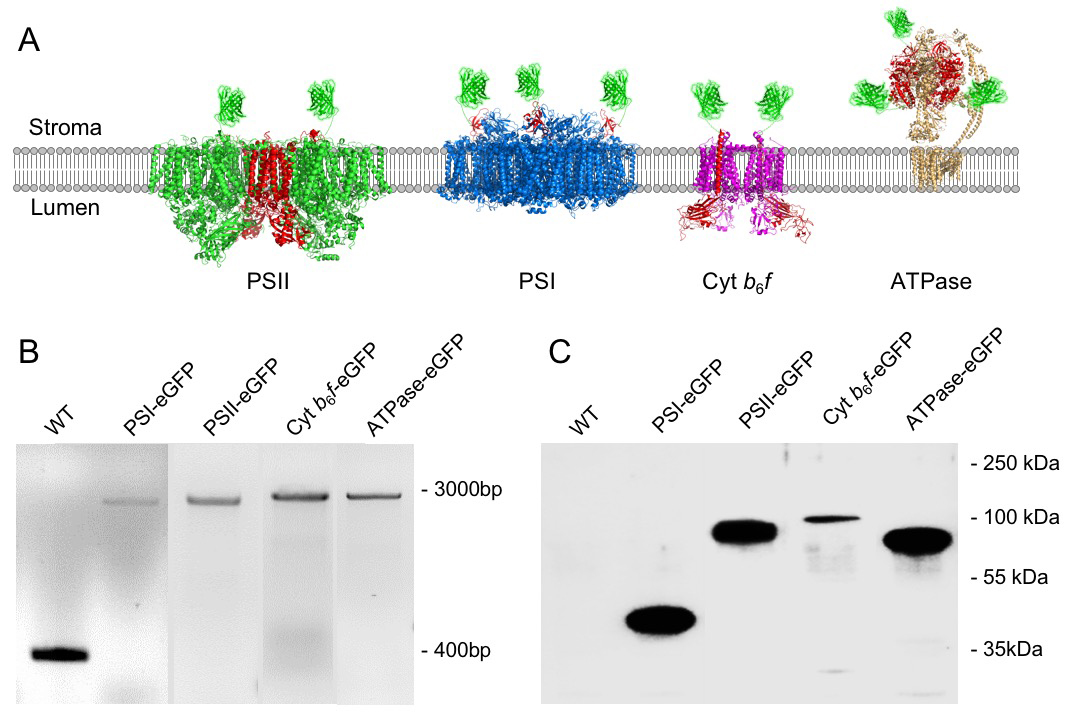


**Supplemental Figure 2. Fluorescence tagging and characterization of photosynthetic complexes in Syn7942.**

(A) Strategy for fluorescence tagging of PSI, PSII, ATPase, and Cyt *b*_6_*f* complexes *in vivo*. The subunits chosen for tagging are PsaE of PSI (three copies per PSI trimer), CP47 (PsbB) of PSII (two copies per PSII dimer), Cyt *f* (PetA) of Cyt *b*_6_*f* (two copies per Cyt *b*_6_*f* dimer), and the subunit β (AtpB) of ATPase (three copies per ATPase). The GFP tags are exposed to the thylakoid stromal membrane surface.

(B) PCR verification of the full segregation of GFP fusion.

(C) SDS-PAGE and immunoblot using anti-GFP antibody show that eGFP is fused to photosynthetic complexes in isolated thylakoid membranes. Immunoblot bands were assigned based on the molecular weights of the tagged subunits.


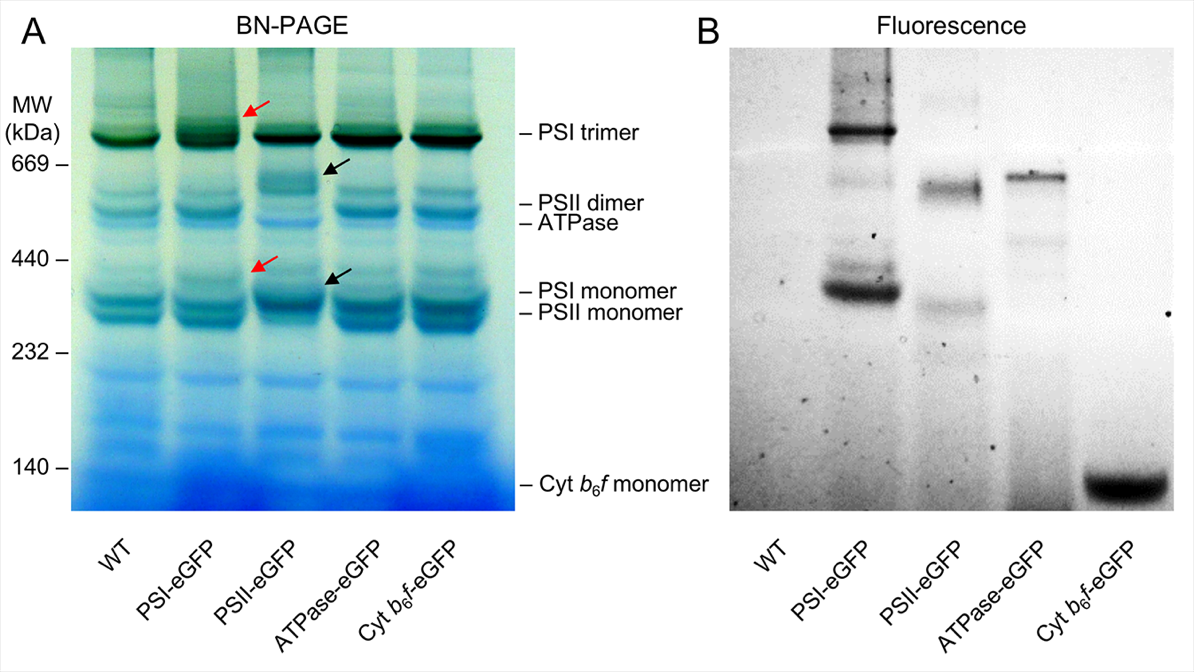


**Supplemental Figure 3. Structural integrity of GFP-tagged photosynthetic complexes.**

(A) BN-PAGE shows GFP fusion does not have detectable effects on the assembly and stoichiometry of photosynthetic complexes in the GFP-tagged strains compared to WT. Shifting of protein bands due to the GFP tagging was indicated in PSI-eGFP (red arrows) and PSII-eGFP (black arrows) strains.

(B) In-gel GFP fluorescence detection of the BN-PAGE gel shown in (A), using an ImageQuant LAS 4000.


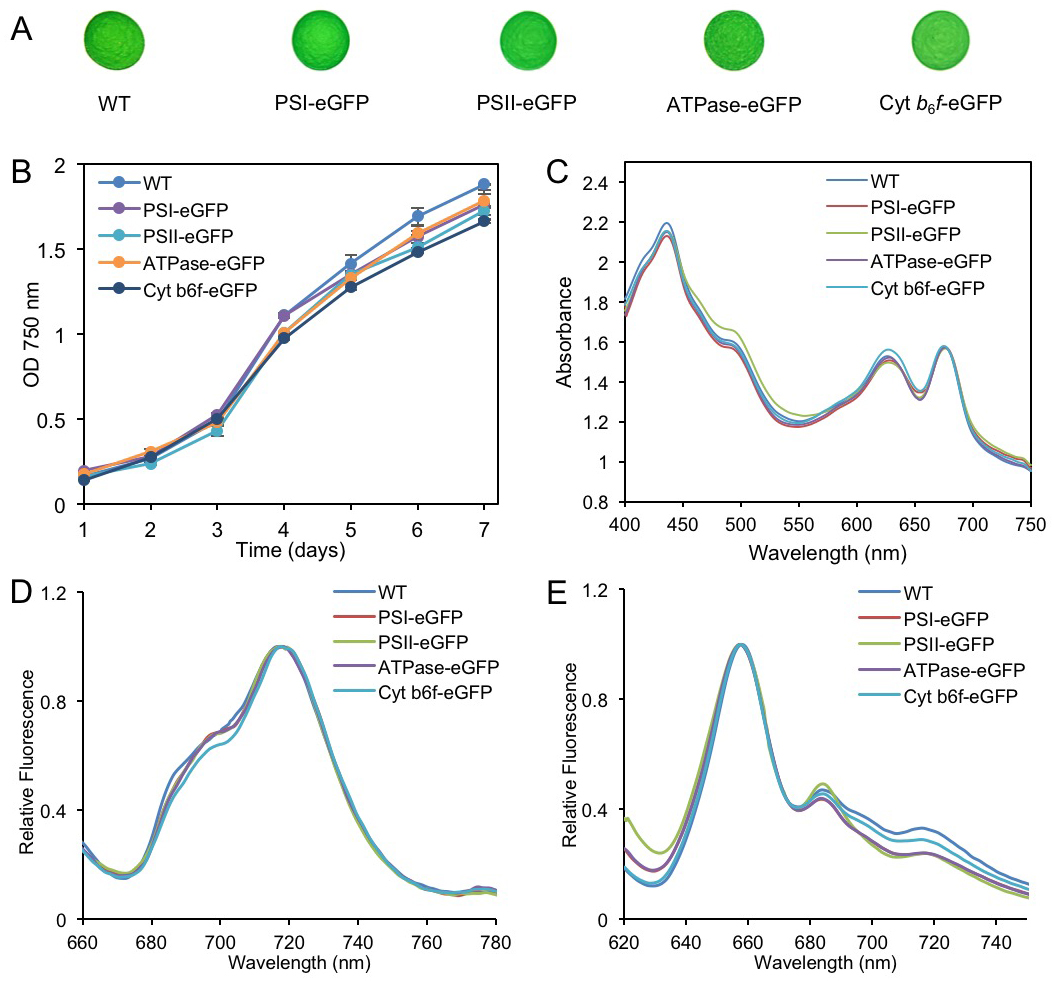


**Supplemental Figure 4. Growth and spectral characterization of WT and GFP-tagged Syn7942 cells.**

(A) Growth of WT and GFP-tagged strains on agar plates. Cells at late exponential phase were diluted to OD750 = 0.1 and spotted on autotrophic agar plates. Plates were incubated for 4 days at 50 µE∙m^-2^∙s^-1^.

(B) Growth of WT and GFP-tagged strains in liquid BG11 medium. Growth was monitored by turbidity of cell suspensions at 750 nm. Results are a mean ± SD of three independent cultures.

(C) Room-temperature absorption spectra of WT and GFP-tagged strains.

(D) 77K fluorescence emission spectra of cells with chlorophyll excitation at 435 nm. Cells were dark-adapted at 30°C before freezing in liquid nitrogen. Spectra were normalized to the long-wavelength emission (mainly from PSI) at 720 nm.

(E) 77K fluorescence emission spectra of cells when excited at 600 nm. Spectra were normalized to the phycocyanin fluorescence emission peak at 650 nm.


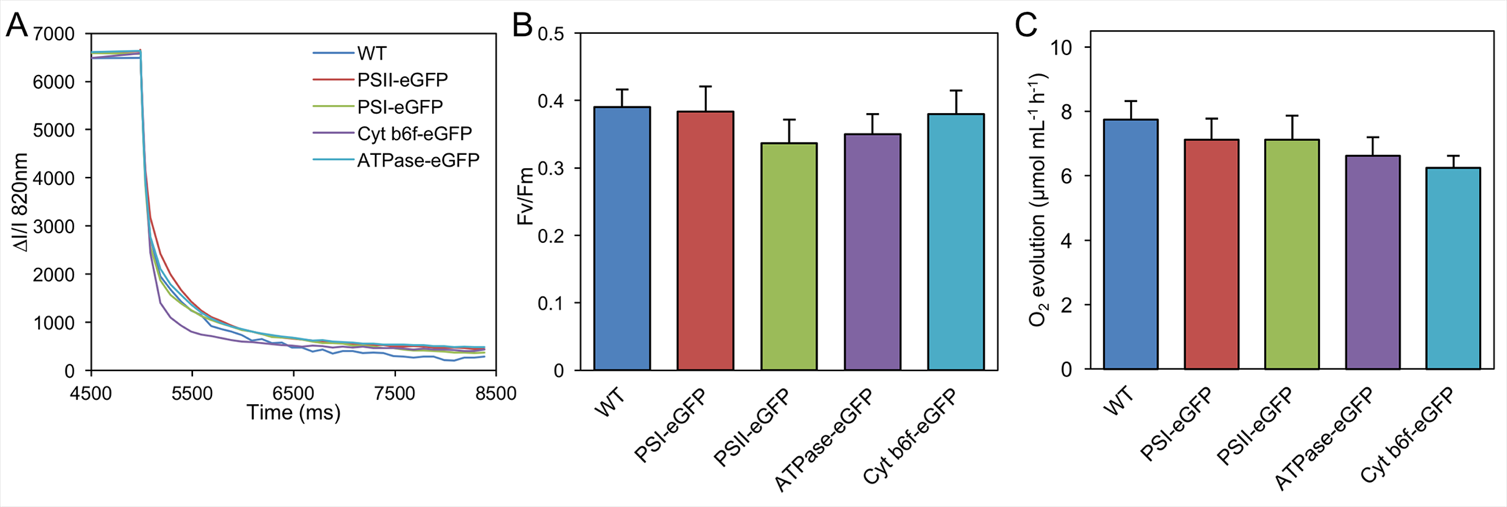


**Supplemental Figure 5. Characterization of P700^+^ re-reduction, PSII activity and electron transport activities of WT and GFP-tagged Syn7942 cells.**

(A) Kinetics of the P700^+^ re-reduction in WT and GFP-tagged strains. P700 re-reduction kinetics were determined at room temperature by analysis of flash-induced absorbance changes at 830 nm. Cells grown at late exponential phase in fresh BG-11 were illuminated by red light (623 nm, 2000 µE∙m^-2^∙s^-1^) with 5 s pulses repeated at 30 s intervals. Each curve represents the mean of 10 accumulations from each of 2 separate aliquots of cells. Curves are normalized to the absorbance of WT.

(B) PSII activity (F_v_/F_m_) of cells grown at exponential phase (*n* = 3).

(C) Rates of oxygen evolution determined in saturated red-light illumination (*n* = 3). Data are represented as mean ± SD.


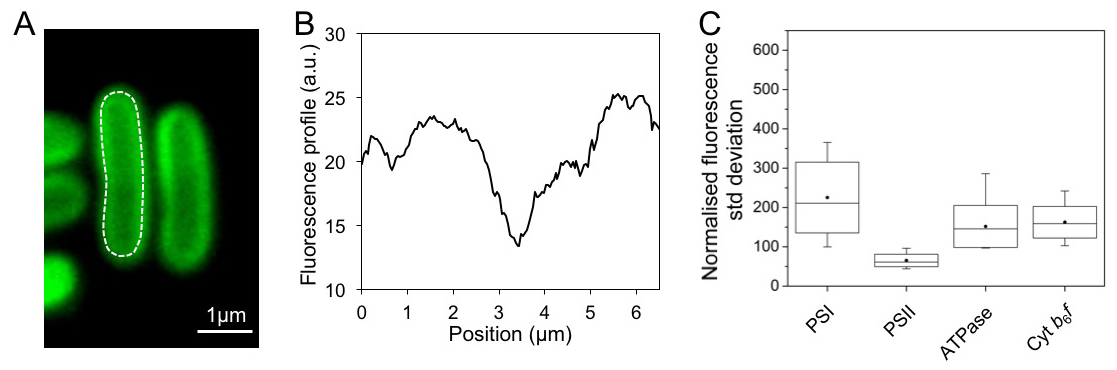


**Supplemental Figure 6. GFP fluorescence profile analysis of the distribution of photosynthetic complexes in the thylakoid membrane.**

(A) An example of GFP fluorescence profile analysis of PSI:eGFP Syn7942 cells.

(B) Normalized fluorescence profiles of the tracing lines in (A).

(C) Fluorescence profile SD of eGFP-fused Syn7942 cells. Data are presented as mean ± SD of the mean.


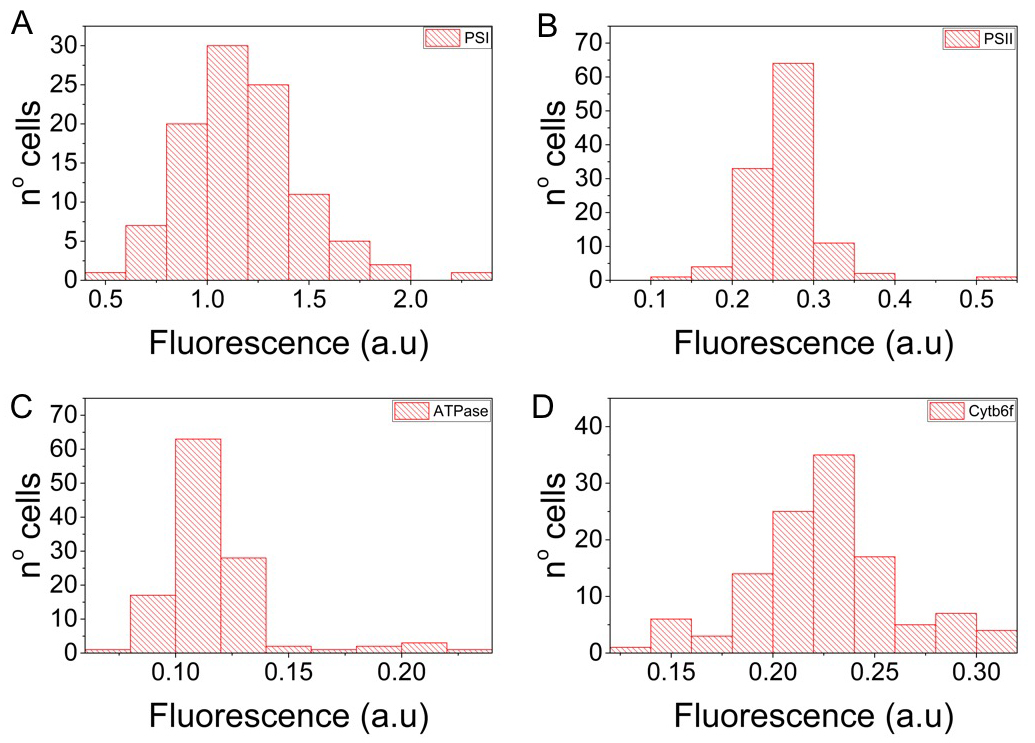


**Supplemental Figure 7. Quantification of total GFP fluorescence intensities per cell of GFP-tagged transformants.**

GFP fluorescence intensity of cells was determined by measuring the total GFP fluorescence of individual cells and extracting background fluorescence of empty regions with the same area. GFP fluorescence intensity per cell was normalized to the cell length and chlorophyll intensity. See Figure 3B.

(A) Histogram of total GFP fluorescence intensities per cell of PSI-eGFP.

(B) Histogram of total GFP fluorescence intensities per cell of PSII-eGFP.

(C) Histogram of total GFP fluorescence intensities per cell of ATPase-eGFP.

(D) Histogram of total GFP fluorescence intensities per cell of Cyt *b*_6_*f*-eGFP.


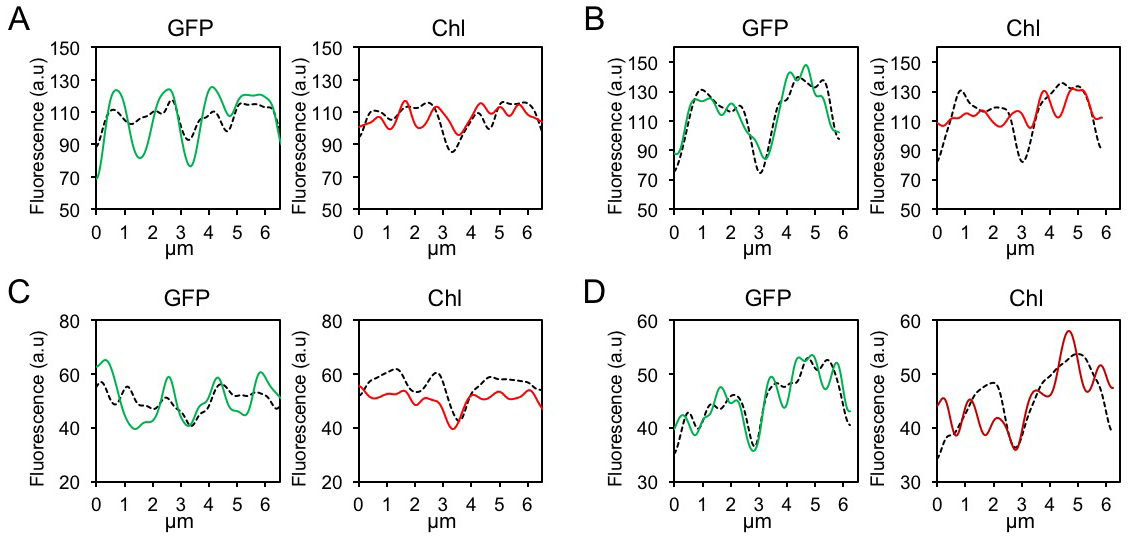


**Supplemental Figure 8. Fluorescence profile analysis of GFP and chlorophyll fluorescence of GFP-tagged transformants before and after red-light treatment.**

Confocal images of these transformants are shown in Figure 6. Fluorescence profiles before red light treatment are shown using black dashed lines. GFP and Chl fluorescence profiles after red light treatment are shown in green and red, respectively. (A) PSI:eGFP. (B) PSII:eGFP. (C) ATPase:eGFP. (D) Cyt *b*_6_*f*:eGFP.

**
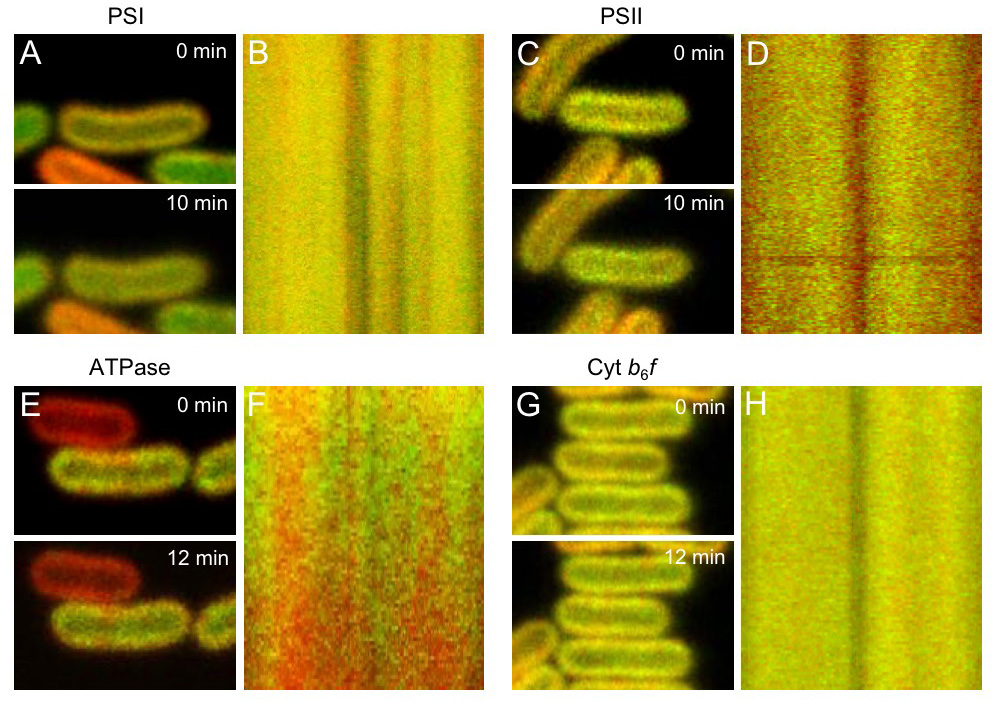
**

**Supplemental Figure 9. Time-lapse confocal microscopy imaging of the distribution and dynamics of photosynthetic complexes without red-light treatment.**

(A, C, E, G) Confocal images of GFP-tagged PSI, PSII, ATPase, and Cyt *b*_6_*f* Syn7942 cells at 0 min and 10 min (for PSI and PSII) or 12 min (for ATPase and Cyt *b*_6_*f*). See Supplemental Movies 5, 6, 7, and 8.

(B, D, F, H) Kymographs of the distribution of photosynthetic complexes, based on the confocal images shown in (A), (C), (E), and (G).


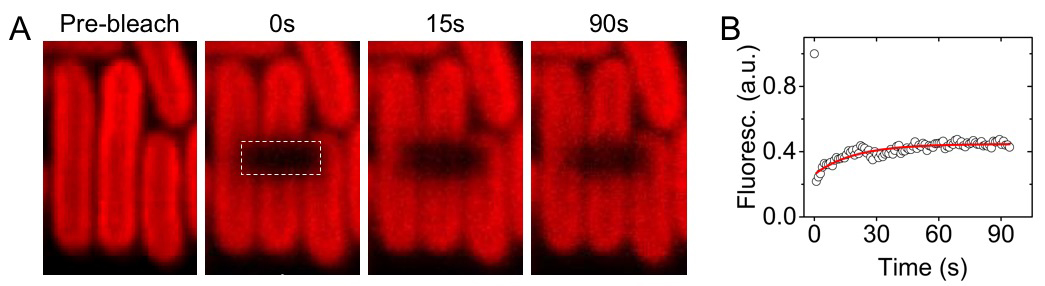


**Supplemental Figure 10. Confocal FRAP analysis of Chlorophyll fluorescence in WT Syn7942, excited with a 633-nm laser.**

(A) Representative FRAP sequence images.

(B) Time course of fluorescence recovery of the bleached cell region (white square in [A[). Fluorescence values are relative to fluorescence prior to the bleach. The recovery of Chl fluorescence is presented as circles and fitted to an exponential function (red line).

**Supplemental Movie 1.** **Dynamics of PSI complexes after red-light treatment.** Data were recorded for 12 min and shown at 15 frames per second. Snapshots and kymograph are presented in Figure 8.

**Supplemental Movie 2.** **Dynamics of PSII complexes after red-light treatment.** Data were recorded for 7 min and shown at 15 frames per second. Snapshots and kymograph are presented in Figure 8.

**Supplemental Movie 3.** **Dynamics of ATPase complexes after red-light treatment.** Data were recorded for 12 min and shown at 15 frames per second. Snapshots and kymograph are presented in Figure 8.

**Supplemental Movie 4.** **Dynamics of Cyt *b*_6_*f* complexes after red-light treatment.** Data were recorded for 12 min and shown at 15 frames per second. Snapshots and kymograph are presented in Figure 8.

**Supplemental Movie 5.** **Dynamics of PSI complexes before red-light treatment.** Data were recorded for 10 min and shown at 15 frames per second. Snapshots and kymograph are presented in Supplemental Figure 9.

**Supplemental Movie 6.** **Dynamics of PSII complexes before red-light treatment.** Data were recorded for 10 min and shown in 15 frames per second. Snapshots and kymograph are presented in Supplemental Figure 9.

**Supplemental Movie 7.** **Dynamics of ATPase complexes before red-light treatment.** Data were recorded for 12 min and shown in 15 frames per second. Snapshots and kymograph are presented in Supplemental Figure 9.

**Supplemental Movie 8.** **Dynamics of Cyt *b*_6_*f* complexes before red-light treatment.** Data were recorded for 12 min and shown in 15 frames per second. Snapshots and kymograph are presented in Supplemental Figure 9.
